# Unraveling the shift in bacterial communities profile grown in sediments co-contaminated with chlorolignin waste of pulp-paper mill by metagenomics approach

**DOI:** 10.3389/fmicb.2024.1350164

**Published:** 2024-03-11

**Authors:** Vineet Kumar, Fuad Ameen, Pradeep Verma

**Affiliations:** ^1^Bioprocess and Bioenergy Laboratory, Department of Microbiology, School of Life Sciences, Central University of Rajasthan, Ajmer, Rajasthan, India; ^2^Department of Botany and Microbiology, College of Science, King Saud University, Riyadh, Saudi Arabia

**Keywords:** high throughput sequencing, metagenome, MiSeq, chloroorganics, *Proteobacteria*, *Thiobacillus*

## Abstract

Pulp-paper mills (PPMs) are known for consistently generating a wide variety of pollutants, that are often unidentified and highly resistant to environmental degradation. The current study aims to investigate the changes in the indigenous bacterial communities profile grown in the sediment co-contaminated with organic and inorganic pollutants discharged from the PPMs. The two sediment samples, designated PPS-1 and PPS-2, were collected from two different sites. Physico-chemical characterization of PPS-1 and PPS-2 revealed the presence of heavy metals (mg kg^−1^) like Cu (0.009–0.01), Ni (0.005–0.002), Mn (0.078–0.056), Cr (0.015–0.009), Pb (0.008–0.006), Zn (0.225–0.086), Fe (2.124–0.764), Al (3.477–22.277), and Ti (99.792–45.012) along with high content of chlorophenol, and lignin. The comparative analysis of organic pollutants in sediment samples using gas chromatography–mass spectrometry (GC–MS) revealed the presence of major highly refractory compounds, such as stigmasterol, β-sitosterol, hexadecanoic acid, octadecanoic acid; 2,4-di-tert-butylphenol; heptacosane; dimethyl phthalate; hexachlorobenzene; 1-decanol,2-hexyl; furane 2,5-dimethyl, etc in sediment samples which are reported as a potential toxic compounds. Simultaneously, high-throughput sequencing targeting the V3–V4 hypervariable region of the 16S rRNA genes, resulted in the identification of 1,249 and 1,345 operational taxonomic units (OTUs) derived from a total of 115,665 and 119,386 sequences read, in PPS-1 and PPS-2, respectively. Analysis of rarefaction curves indicated a diversity in OTU abundance between PPS-1 (1,249 OTUs) and PPS-2 (1,345 OTUs). Furthermore, taxonomic assignment of metagenomics sequence data showed that *Proteobacteria* (55.40%; 56.30%), *Bacteoidetes* (11.30%; 12.20%), and *Planctomycetes* (5.40%; 4.70%) were the most abundant phyla; *Alphproteobacteria* (20.50%; 23.50%), *Betaproteobacteria* (16.00%; 12.30%), and *Gammaproteobacteria* were the most recorded classes in PPS-1 and PPS-2, respectively. At the genus level, *Thiobacillus* (7.60%; 4.50%) was the most abundant genera grown in sediment samples. The results indicate significant differences in both the diversity and relative abundance of taxa in the bacterial communities associated with PPS-2 when compared to PPS-1. This study unveils key insights into contaminant characteristics and shifts in bacterial communities within contaminated environments. It highlights the potential for developing efficient bioremediation techniques to restore ecological balance in pulp-paper mill waste-polluted areas, stressing the importance of identifying a significant percentage of unclassified genera and species to explore novel genes.

## 1 Introduction

The pulp-paper mill (PPM) stands as a significant industrial segment that extensively utilizes large quantities of lignocellulosic feedstock and freshwater throughout the paper manufacturing procedure. Although this industrial sector contributes a significant role to the world’s economy, this industry in particular is known to be one of the largest producers of wastewater worldwide emanated during subsequent industrial operations (Bui et al., 2022; Namdarimonfared et al., 2023). From the different stages of the paper-making process, wastewater from the bleaching process is probably the major problem of PPMs (Oke et al., 2017; Kumar et al., 2020; Coimbra et al., 2021). It is estimated that freshwater ranging from 273 to 450 m^3^ is essential to make 1 tonne of paper in PPMs and about 60–300 m^3^ of bleached wastewater is generated (Mittar et al., 1992). Based on a survey report, approximately 3 billion cubic meters of wastewater discharged annually from PPMs in the 21st century (FPAC, 2009). There are about 900 PPMs spread across different states of India (Central Pulp Paper Research Institute, 2021).

Bleaching of pulp by chlorine-based chemicals to remove the characteristic brown color of lignin for whitening pulp as a part of pulp processing in PPMS is still practiced in India (Kaur et al., 2018; Rajput et al., 2020) and many other developing countries (Liang et al., 2021). As a consequence, a myriad of chloroorganic compounds, such as chlorolignin, chlorophenols, chlorohydrocarbons, chlorlignosulphonic acids, chlorinated resin acids, dibenzo-*p*-dioxin, and dibenzofuran, which are formed inadvertently as adsorbable organic halides (AOX), are discharged in the bleaching wastewater stream (Deshmukh et al., 2009; Zhang et al., 2020; Kumar and Verma, 2023). Bleach wastewater exhibits high strengths of chemical oxygen demand, suspended solids, dissolved lignin, color, and a blend of various organic compounds (Sharma et al., 2014; Kumar A. et al., 2021; Liang et al., 2021). Recent reports have brought to light the presence of per- and polyfluoroalkyl substances (PFASs) in the effluent wastewater of PPMs (Chow and Foo, 2023). In addition, inorganic chemical contaminants including various heavy metals have also been concomitantly discharged in bleached wastewater (Bhatti et al., 2021; Singh et al., 2021). Organic substances, including AOX, have become a significant and growing concern. They are characterized by their remarkable persistence in open environments over extended periods, high mobility, and the ability to traverse vast distances within ecosystems. Eventually, these substances reach the food chain and accumulate in the adipose tissues of the human body (Costigan et al., 2012; Castro et al., 2018; Balabanič et al., 2021). This presents not only a serious health hazard but also inflicts irreparable damage to pristine ecosystems, biodiversity, soil quality, and water pollution. Moreover, non-degradable heavy metals and organometallic pollutants also pose an imminent threat to human health (McElroy et al., 2011; Mitra et al., 2022).

The disposal of bleached wastewater from PPMs into freshwater bodies, prior to the implementation of wastewater treatment processes, has led to the accumulation of a substantial volume of solids in the sediments of the terrestrial ecosystem (Yadav and Chandra, 2018; Tripathy et al., 2022). These deposited solids typically include various components, such as wood fibers, organochlorine compounds, heavy metals, papermaking fillers, pitch, lignin by-products, and ash. Previous studies have reported that wastewater discharged from the PPMs during subsequent industrial operations at the primary and secondary levels still retain an extreme load of multifaceted pollutants which significantly deteriorate the quality of the environment if discharged back into the environment (Orrego et al., 2010, 2019; Yuliani et al., 2019; Sharma et al., 2021a). However, there is still a lack of detailed knowledge regarding the specific nature of the pollutants discharged from PPMs.

It has been reported that indigenous microbial communities, predominantly composed of bacteria, play pivotal roles in the recycling of organic matter, including their involvement in the biodegradation of organic compounds, and contribute to the sustainable eco-restoration of contaminated sites (Guo et al., 2017; Selvi et al., 2021; Muthukumar et al., 2022; Chen et al., 2023). Wang et al. (2023) demonstrated that colonized functional microbes carried by agro-industrial waste played an important role in the co-pollutant removal. Prakash et al. (2021) highlighted the bioreduction of hexavalent chromium Cr(VI) to trivalent chromium Cr(III) by bioelectrokinetic techniques. Next-generation sequencing (NGS)-data analysis revealed the presence of *Proteobacteria, Firmicutes, Bacteroidetes, Actinobacteria*, and *Planctomycetes* in the bio-electrokinetic system. Proteobacteria are responsible for the bioreduction of Cr(VI) by the formation of FeS particles. Yang X. et al. (2023) explored the environmental pollution behavior/fate of ammunition soil and microbial remediation of trinitrotoluene (TNT), such as 2-amino-4,6-dinitrotoluene (2-ADNT), 4-amino-2,6-dinitrotoluene (4-ADNT), and 2,4-diamino-6-nitrotoluene (2,4-DANT) and its intermediates. The abundance of Sphingomonadaceae showing key tolerance/degradation TNT was significantly upregulated. However, organic compounds and heavy metals can impact the growth and survival of microbes in tailings soil and sludge, leading to variations in community structure and diversity (Yergeau et al., 2012; Kumar and Chandra, 2020; Tong et al., 2021; Mapelli et al., 2022; Lee et al., 2023; Yang L. et al., 2023). The contamination of chlorolignin compounds and heavy metals can have adverse effects on the diversity, survival, growth, and other ecological functions of soil microbial communities. An exploration of bacterial structures and community functions can provide insights into how microbial communities respond to variations in contaminant levels within an open environment. Thus, the detailed information on the profile of indigenous bacterial communities’ structure and functions surviving in chlorolignin waste contaminated sites will provide insight into possible strategies for decontamination and eco-restoration of polluted sites employing bacterial communities.

To date, only an inadequate number of microbial agents have been identified that can effectively degrade and treat wastewater or pollutants discharged from PPMs (Fernandes et al., 2014; Sonkar et al., 2019; An et al., 2021; Hajdu-Rahkama and Puhakka, 2022; Yue et al., 2023). This scarcity of microbial agents capable of treating paper mill wastewater may be attributed to challenges in isolating and culturing these microbial species under controlled conditions. The culturing technique has been broadly employed as a direct and efficient technique for culturing and characterizing the microbial community thriving in complex environments (Tripathi et al., 2022). However, it’s worth noting that a significant portion of microbes in natural environments cannot be cultured in a laboratory’s artificial medium, and the diversity of uncultured microbes is quite extensive.

Over the last decade, an array of molecular methods, such as terminal restriction fragment length polymorphism (T-RFLP) (Osborn et al., 2000), denaturing gradient gel electrophoresis (DGGE) (Muyzer and Smalla, 1998), and Fluorescent *in situ* hybridization (FISH) (Moter and Göbel, 2000), has greatly promoted our understanding of the microbial community. However, for a multifaceted environment with vast genetic diversity, these conventional methods fall short of providing a comprehensive view of the bacterial community, offering only limited information about microbial populations (Ranjard et al., 2000; Prada et al., 2022). In current decades, thanks to the progress in second-generation sequencing and computational biology methods, it has become feasible to obtain insights from previously uncultured beneficial microbial communities. It’s now possible to scrutinize changes in microbial communities within environmental samples at the molecular level with an unprecedented level of detail through metagenomic analysis using NGS technology.

Metagenomics, a revolutionary robust approach based on high-throughput sequencing (HTS), serves as a powerful tool to enable the accurate mining of novel microbes and functional genes from the environmental samples without identifying them individually (Han et al., 2020; Fan et al., 2022). In addition, it can overwhelm the shortcomings of conventional microbial community analysis methods, and unveil the microbial community interactions and their function in contaminated environments (Abia et al., 2018; Qian et al., 2022). Limited studies have been directed to explore the microbial community structure and composition grown in contaminated environments of pulp paper mill wastewater (PPMW). Sharma et al. (2021b) profiled the microbial community abundance and their structure grown in the PPMW, which contained a blend of toxic metals and organic chemicals, through a metagenomics approach. Tripathi et al. (2022) characterized the wide array of persistent organic pollutants (POPs), which influence the culturable and unculturable bacterial communities, in sludge of PPMs discharged after secondary treatment. In a recent study by Sharma et al. (2023), they quantified the microbial communities by 16S rRNA analysis thriving in activated sludge of PPMs containing lignin and chlorophenol. While various efforts have been made to clean up the environment polluted by pulp and paper mill discharge by focusing on indigenous microorganisms, the quest for an efficient and cost-effective solution continues (Sonkar et al., 2019; Kumar et al., 2022). Surprisingly, there have been no initiatives to investigate the profile of the bacterial communities within the sediments that accumulate over time in the ecosystem receiving discharges from PPMs. The exploration of how indigenous microbial communities respond can provide valuable insights for researchers in their search for eco-friendly solutions to combat soil and/or water pollution.

The present study examined variations in the profile of bacterial community diversity and composition grown within two sediment samples. These changes are believed to be the driving force behind the improvement in pollutant conditions, ultimately fostering biological succession and bioremediation. This, in turn, holds promise for the ecological restoration of sites burdened by a heavy load of wastewater pollutants discharged from PPMs. The specific objectives of this study were to: (1) analyze the impact of discharged wastewater and their pollutants on the bacterial community in the sediment samples; (2) detect and characterize the broad range of refractory organic pollutants, extracted with two different solvents, by gas chromatography–mass spectrometry (GC–MS) technique; (3) identify the prevalent indigenous bacterial community, unveiling the microbial niche within this contaminated environment by metagenomic approach; and (4) discuss the relationship between the bacterial community and pollutants rich environment. The findings presented in this study offer valuable insights that contribute to the comprehension of the mechanisms involved in both *ex situ* and *in situ* bioremediation, facilitating the safe disposal of such waste.

## 2 Materials and methods

### 2.1 Chemicals and reagents

In this study, HPLC grade organic solvents such as ethyl acetate, dichloromethane (DCM), and methanol that were obtained from Merck (Merck India) and had a purity exceeding 99%. Ethyl acetate and DCM were employed for the recovery of organic pollutants from the sediment samples. Additionally, other chemicals including BSTFA (N,O-bis(trimethylsilyl)trifluoroacetamide), dioxane, and pyridine, employed in the derivatization process, were sourced from Sigma-Aldrich (Saint Louis, MO, USA). For the preparation of digestion mixtures to extract heavy metals from the sediments, concentrated hydrochloric acid (HCl; 37%) and nitric acid (HNO_3_; 69.5%) were procured from HiMedia Laboratories (Maharashtra, India).

### 2.2 Instrumentation

Throughout this study, digital pH meter (315, Systronics, India); conductivity meter (Orion StarTM A212, Thermo Scientific^™^, FL, USA), ion electrodes (Orion auto analyzer model- 960, USA); Rotavapor (Heidolph, Germany); inductively coupled plasma mass spectrometry (ICP-MS; Nexion Ions, Perkin Almer); GC–MS (Agilent 8890/5977B Series; Agilent Technologies, Inc) was utilized.

### 2.3 Collection of samples

**Figure 1:**
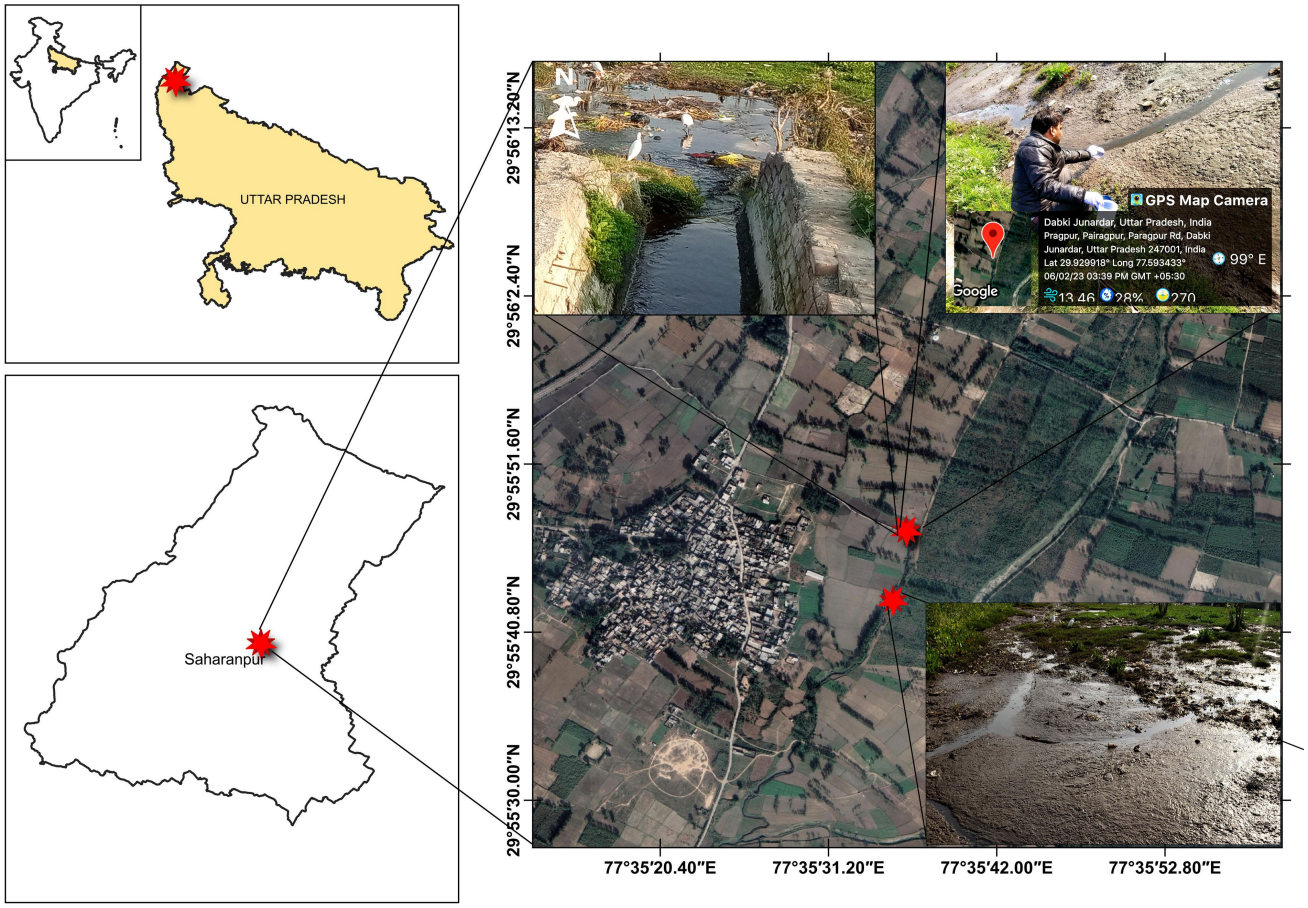
The map indicating the geographical location of sample collection site from two sites situated in Paragpur village, Saharanpur, Uttar Pradesh, India. The star in red color represents the two different sampling points where from the samples were collected.

Star Paper Mill Ltd., an integrated paper mill Established in 1938, is one of the leading sellers of industrial and craft paper, printing paper, writing paper, packaging, and cultural papers located in Saharanpur, Uttar Pradesh, India. The mill employs woody raw feedstock, including poplar, eucalyptus, and veneer chips, for the production of a diverse range of high-grade papers (Dagar et al., 2022). In the paper manufacturing process, a Kraft method is utilized to make pulp from wood chips, followed by chlorine bleaching to produce white paper. The mill is equipped with a comprehensive effluent treatment plant that employs the activated sludge process, consisting of a primary clarifier, aeration basin, secondary clarifier, and sludge dewatering. After secondary treatment, this mill discharges its wastewater through a covered canal in the open area which is located in the Paragpur village of the Saharanpur District, India (Figure 1). Due to the water scarcity, the discharged wastewater is used by the farmers of this village to irrigate agricultural lands, which is a public practiced in developing nations including India (Medhi et al., 2011; Singh et al., 2012; Mishra et al., 2023). In a 2012 field study by Kumar and Chopra, wastewater from the Star Paper Mill in Saharanpur (Uttar Pradesh) was utilized in various concentrations as irrigates for the cultivation of *V. radiata*. The study found that the mill’s effluent led to a reduction in soil moisture content, water-holding capaciy, and bulk density. Simultaneously, it resulted in an increase in pH, electrical conductivity (EC), chloride (Cl^−^), sulfate (SO_4_^2−^), phosphate (PO_4_^3−^), nitrate (NO_3_^2−^), total Kjeldhal nitrogen (TKN), calcium (Ca^2+^), potassium (K^+^), sodium (Na^+^), carbonate (CO_3_^2−^), bicarbonate (HCO_3_^−^), organic carbon, and various metals in the soil (Kumar and Chopra, 2012). The discharged site was located only about 2 km away from the mill and is one of the highly contaminated areas of Saharanpur, as reported by earlier researchers (Kumar et al., 2019; Singh et al., 2021). The mill discharged its wastewater after the paper manufacturing process through a covered canal which is finally mixed with the water of the Hindon River which finally Mixed with Yamuna River’s water (Sharma et al., 2021c; Dagar et al., 2022). The effluent discharged site selected for sample collections was denoted as Site-1, while the second site (Site-2) was 1,000 meters far away from Site-1, as shown in Figure 1. Triplicate sediment samples were randomly collected in a sterile plastic container (1 L) using an excavator bucket from each sampling sites at a depth of 0–10 cm. The depth of soil sampling is significantly affected abundance and diversity of microbial community (Böer et al., 2009; Mendes and Tsai, 2014; Fu et al., 2023). The selection of the 0–10 cm depth range for collecting sediment samples is often based on the findings of earlier researchers (Zhao et al., 2021; Beule et al., 2022). Randomly collected samples were mixed to get one composite sample from each site, Site-1, and Site-2, and were designated as PPS-1 and PPS-2, respectively. The collected sediment samples were maintained at a temperature of 4°C and promptly transported in ice packed box to the laboratory for subsequent investigation. A temperature of 4°C is recommended to inhibit bacterial growth and decelerate metabolic processes, ensuring a dependable metagenomic analysis within 24 h of collection (Vandeputte et al., 2017).

### 2.4 Physico-chemical characterization of samples

Samples taken from two different sites (Site-1 and Site-2), as depicted in Figure 1, were analyzed to determine the physico-chemical load of various pollutants including pH, electrical conductivity (EC), potassium (K^+^), sodium (Na^+^), total organic carbon (TOC), etc., as per the standard methods described earlier (Kumar and Chandra, 2020 Kumar V. et al., 2021). EC and pH measurements were performed on 1:2.5 sediment-water suspensions and measured by conductivity meter and Digital pH meter, respectively. The total concentrations of sodium (Na^+^), chloride (Cl^−^), and sulfate (SO_4_^2−^) were quantified based on the procedure outlined by Kalra and Maynard (1991). Total phenol content in was quantified employing the 4-aminoantipyrine reaction method (Ettinger et al., 1951). Lignin content was quantified and estimated using the methodology described by Pearl and Benson (1990). The concentrations of chlorophenol in the samples were determined following standard procedures (Choudhary et al., 2012).

### 2.5 Heavy metal analysis

The heavy metal contents in the sediment samples were estimated via ICP-MS, as outlined by Kumar and Chandra (2020). For heavy metal determination, sediment samples underwent digestion using the HNO_3_-H_2_O_2_ digestion method (3050B of USEPA, 1996). After digestion, the volume was adjusted with ultra-pure deionized water and filtered by Whatman filter paper No. 42 prior to analysis. Subsequently, the ICP-MS method was utilized to ascertain the overall concentration of various heavy metals in filtered and digested samples.

### 2.6 Detection and characterization of organic pollutants

#### 2.6.1 Sample pre-processing and solid -liquid extraction

A pre-weighed 25 g sediment sample was weighed and placed into the Erlenmeyer flask (250 mL), and 100 mL of distilled water was added to each flask separately. Thereafter, the mixture was continuously agitated at room temperate in a rotatory incubator shaker for 48 h to mix vigorously, and the sludge suspension was allowed to stand still for 6 h. After this, the suspension was filtrated by passing through the Whatman filter paper No. 42. Next, the filtrate was acidified with H_2_SO_4_ 1 M up to pH 2.0. Subsequently, acidified filtrate was employed to extract the organic compounds with two different solvents (separately) namely ethyl acetate and DCM, as per the standard liquid–liquid extraction procedure (Chandra et al., 2017). The choice of ethyl acetate and DCM as solvents for liquid–liquid extraction in sample pre-processing is often based on their selectivity and compatibility with the classes of phenolic compounds typically found in sediment samples (Möder et al., 1997; Kumar, 2014; Azzam and Hazaimeh, 2021; Sharma et al., 2021a). Briefly, 50 mL of acidified supernatant was transferred into a 500 mL separatory funnel. Subsequently, an equal volume of organic solvent was added, and the mixture was vigorously shaken for 15 min. Thereafter, the organic phase was separated from the aqueous phase. This extraction step was repeated successively thrice to extract the maximum number of organic compounds. Subsequently, the organic phases collected from separating funnel in a 250-mL beaker were combined, evaporated up to near dryness using a vacuum rotary evaporator at ≤40°C, and then and dehydrated over anhydrous sodium sulfate to eliminate water traces. Finally, the dried residues were reconstituted in methanol (2.0 mL), filtered through 0.22-μm Millipore syringe filters. The final dissolved extracts were analyzed by GC–MS. For GC–MS analysis, a silylation reagent mixture consisting of 100 μL BSTFA, 50 μL dioxane, and 50 μL pyridine, was added to the extracts (Sonkar et al., 2019).

#### 2.6.2 Gas chromatography-mass spectrometry analysis

GC–MS analysis of extracts was performed with Gas Chromatograph (Agilent 8890; Agilent Technologies, Inc) quipped with autosampler coupled to 5977B Series double-quadrupole Mass Selective Detector (MSD) system (Agilent Technologies, Inc) operated in full-scan mode using a mass range of 30–550 amu to obtain their mass spectrogram. The mass range of 30–550 amu was chosen to encompass a broad spectrum of high molecular weight organic compounds commonly found in environmental samples (Medeiros, 2018; Kumar V. et al., 2021, 2022). This range was considered to cover a diverse array of organic pollutants, including chlorinated compounds discharged in wastewater that may be present in sediment matrices. In the analysis, a 1.0 μL aliquot of the extract was used. Organic compounds were separated using a DB-5 MS capillary column (0.25 mm × 60 m × 0.25 μm) (Agilent Technologies, Inc) contains 5% phenyl-methylpolysiloxane as a stationary phase. The temperature of the injector and detector were worked at 280°C. The temperature of the GC oven was initially programmed as follows; 70°C (held 2 min), 6°C min^−1^ up to 230°C (2 min); 6°C min^−1^ up to 280°C (hold time: 20 min). Helium with a purity of 99.99% served as the carrier gas, and the column flow was sustained at a rate of 1.1 mL min^−1^. The total chromatographic run was 60 min. For instrument control, data acquisition, and evaluation MassHunter GC/MS Acquisition 10.1.49 was used (Agilent Technologies, Inc). The identification of the organic compounds eluted from the GC was carried out by analyzing their mass spectra and comparing them to entries in the National Institute of Standards and Technology (NIST) mass spectrum library search for matching and identification.

### 2.7 Characterization of bacterial communities in sediments

#### 2.7.1 Preparation of samples for NGS analyses: DNA extraction and amplification

Metagenome was recovered from pre-weighted 1 g of representative homogenized sediment samples under sterile conditions using the NucleoSpin^®^ Soil kit (Macherey-Nagel, GmbH & Co. KG) following the manufacturer’s instructions. The quantity and quality of metagenome were assessed by measuring the absorbances at A_260_ nm and A_280_ nm using a NanoDrop 2000 Spectrophotometer (ThermoFisher Scientific, Massachusetts, USA). Subsequently, gel electrophoresis was conducted using a 1.2% agarose gel as an additional quality control step for the extracted DNA. This was undertaken to identify and evaluate the presence of any contaminants that could potentially interfere with the activity of enzymes necessary for subsequent analyses. The criteria typically used to assess the quality of DNA on a gel are size estimation, band intensity, single band presence, band sharpness and resolution, and absence of RNA contamination and high molecular weight smearing. Thereafter, the V3 and V4 hypervariable regions of the 16S rRNA gene were amplified with the universal bacterial forward primers; 341F (5′-CCTACGGGNGGCWGCAG-3′) and reverse primer; 805R (5′-GAC- TACHVGGGTATCTAATCC-3′). The PCR (Polymerase Chain Reaction) was conducted using a Perkin Elmer Thermocycler following this procedure: initial denaturation at 98°C for 3 min, followed by 27 cycles of denaturation at 98°C for 15 s, annealing at 50°C for 30 s, and extension at 72°C for 30 s. The process concluded with a final extension step at 72°C for 5 min. The first amplicons were recovered with 1.2% agarose gel (2 μL sample) and then purified and quality checked again, then the sequencing library was built.

#### 2.7.2 Library construction, and high-throughput sequencing

The Illumina paired-end multiplexed sequencing library was constructed using the Nextera XT Index kit (Illumina Inc.) following the standard protocol (part#15044223 Rev. B.). The amplicon library was then subjected to purification by AMPureXP beads and quantified using Qubit^®^ 3.0 Fulorometer (Thermo Fisher Scientific, Waltham, USA). The amplified library underwent analysis using a 4,200 Tape Station System (Agilent Technologies, Santa Clara, USA) with D1000 Screen tape for quality control, following the manufacturer’s instructions. The Qubit fluorometric concentration for the libraries was determined. Following the determination of the mean peak size from the Tape Station Profile, the libraries were loaded onto the MiSeq platform (MiSeq PE300; Illumina, San Diego, USA) at an appropriate concentration (10–20 pM) for cluster generation. A 2 × 300 paired-end sequencing run was performed on the MiSeq sequencer to generate the raw reads.

#### 2.7.3 Illumina data processing and microbiota characterization

Firstly, the quality of the raw sequencing data generated by the Illumina MiSeq sequencing platform was processed, and quality trimming (Q N 30) and length trimming were conducted to obtain high quality reads for subsequent analysis. To ensure data reliability, low-quality reads—those with more than 10% of quality threshold (QV) <20 Phred scores—were eliminated using the Trimmomatic (v0.38) software^1^ (Bolger et al., 2014). The high-quality (QV > 20), paired-end reads were used for read assembly. The filtered metagenomic reads were used for taxonomical assignment. Operational taxonomic unit (OTU) analysis of high quality paired-end FASTQ reads from Illumina sequencing was performed using the software package Quantitative Insights into Microbial Ecology 2 program (QIIME v1.8.0^2^) (Caporaso et al., 2010). The closed-reference OTU picking method was employed, and sequences were searched against the Greengenes database (version 13_8).^3^ Furthermore, in this study, the UCLUST classifier was employed to group high-quality sequencing data into OTUs with a 97% similarity threshold within the QIIME platform. The taxonomic classification of each 16S rRNA gene sequence was performed using the Ribosomal Database Project (RDP) Classifier algorithm^4^, and the representative OTU sequences were compared to those in the Silva (SSU132) 16S rRNA database. Alpha diversity indices were obtained using the QIIME, including Shannon and Simpson diversity indices (Feranchuk et al., 2018). Stacked bar plots and rarefaction curves were generated using the R package Phyloseq (McMurdie and Holmes, 2013) and Microsoft Excel (Microsoft Office^®^ version 2018). Rarefaction curves were created utilizing the Shannon and Observed-OTU indices through QIIME (version 1.7.0) and visualized using R software (version 2.15.3). Venn diagrams were generated to depict the number and similarity of OTUs using a Venn plotter available at http://bioinformatics.psb.ugent.be/webtools/Venn/. The abundance distribution of dominant bacterial genera among all samples was displayed in the species abundance heat map. The OTU -heat map displays raw OTU count per sample, where the counts are colored based on the contribution of each OTU to the total OTU count present in that sample. Heatmap analysis, focusing on the most abundant OTUs in the entire libraries, was performed using the heatmap.2 function within the R package gplots (version 3.1.0).^5^ The taxonomic composition of PPS-1 and PPS-2 was visualized using a Krona chart generated through the Krona web application, available at https://github.com/marbl/Krona/wiki. A workflow that represents the major steps associated with bioinformatic analysis of high throughput sequence data is depicted in Supplementary Figure S1.

## 3 Results

### 3.1 Physico-chemical analysis of sediment samples

The mean values of major physico-chemical parameters in sediment samples collected from Site-1 and Site-2 were determined, summarized in Table 1. The results revealed that PPS-1 had high pH (8.07 ± 0.01) and EC (2767.5 ± 1597.816 μS cm^−1^). PPS-1 also had significant content of sulfate, Cl^−^, and phosphate of approximately 24.056 ± 0.005, 1891 ± 2.00, and 0.761 ± 0.010 mg kg^−1^, respectively. In addition, very high concentrations of the other chemical indicators like total phenol (352.333 ± 1.527 mg kg^−1^), lignin (1161.333 ± 60.351 mg kg^−1^), chlorophenol (618 ± 3 mg kg^−1^), total organic carbon (11.601 ± 0.005 mg kg^−1^), and total carbon (42.741 ± 8.7023 mg kg^−1^), were detected in the PPS-1. As shown Table 1, the pH and EC of PPS-2 samples presented a slight decrease with value of 7.633 ± 0.057 and 722 ± 2 μS cm^−1^, respectively, collected from Site-2. The value of sulfate (23.458 ± 0.005 mg kg^−1^) and Cl^−^ (1753 ± 2.645 mg kg^−1^) were slightly lower in the PPS-2 sediment sample. However, the phosphate concentration (0.824 ± 0.005 mg kg^−1^) was higher compared to PPS-1. Additionally, it was observed that, lignin (1102 ± 1 mg kg^−1^), total phenol (324.333 ± 0.577 mg kg^−1^) and chlorophenol (522 ± 2 mg kg^−1^) exhibited lower concentrations at Site-2 when compared to Site-1.

**Table 1:** Physico-chemical characteristics of sediments samples collected from two different sites polluted with chlorolignin contaminants of pulp-paper mills.

Parameters	Unit	Sample	Min	Max	Mean	SD
pH	–	PPS-1	8.06	8.08	8.07	0.01
		PPS-2	7.6	7.7	7.633	0.057^***^
EC	μS cm−^1^	PPS-1	2767.5	2767.45	2767.5	1597.816
	μS cm−^1^	PPS-2	720	724	722	2^*^
Sulfate	mg kg^−1^	PPS-1	24.052	24.062	24.056	0.005
	mg kg^−1^	PPS-2	23.453	23.463	23.458	0.005^ns^
Chloride	mg kg^−1^	PPS-1	1889	1893	1891	2.00
	mg kg^−1^	PPS-2	1751	1756	1753	2.645^**^
Phosphate	mg kg^−1^	PPS-1	0.749	0.769	0.761	0.010
	mg kg^−1^	PPS-2	0.819	0.829	0.824	0.005^**^
Sodium	mg kg^−1^	PPS-1	455	459	456.666	2.081
	mg kg^−1^	PPS-2	385	387	386	1^*^
Chlorophenol	mg kg^−1^	PPS-1	615	621	618	3
	mg kg^−1^	PPS-2	520	524	522	2^*^
Lignin	mg kg^−1^	PPS-1	1,125	1,231	1161.333	60.351
	mg kg^−1^	PPS-2	1,101	1,103	1,102	1^*^
Total Phenol	mg kg^−1^	PPS-1	351	354	352.333	1.527
	mg kg^−1^	PPS-2	324	325	324.333	0.577^*^
Total organic carbon	mg kg^−1^	PPS-1	11.596	11.606	11.601	0.005
	mg kg^−1^	PPS-2	2.355	2.357	2.354	0.003^***^
Total carbon	mg kg^−1^	PPS-1	42.741	42.741	42.741	8.7023
	mg kg^−1^	PPS-2	38.74	38.755	38.745	0.008^**^

SD, Standard deviation; Min, Minimum; Max, Maximum; EC, Electrical conductivity; Asterisks indicate significant differences between the PPS-1 and PPS-2. Student’s *t*-test, two-tailed: *Highly significant at *p* < 0.001, **Significant at *p* < 0.01, ***Less significant at *p* < 0.05, ns Non-significant at *p* > 0.05.

### 3.2 Heavy metal content

The average concentration of analyzed metals in the sediment samples is shown in Supplementary Table S1; certain metals, such as titanium (99.792 ± 0.016 mg kg^−1^), aluminum (3.477 ± 0.006 mg kg^−1^), and iron (2.127 ± 0.002 mg kg^−1^), displayed a high concentration in PPS-1. Out of 17 analyzed metals in PPS-1, vanadium and molybdenum were found to be below detectable limit. In contrast to PPS-1, the values of various heavy metals determined in the PPS-2 sample were slightly lower, as tabulated in Supplementary Table S1. Therefore, further investigations are imperative to characterize the organic pollutants sequestered in the sediment samples. Profiling of organic pollutants in sediment samples will be useful for the development of effective and novel bioremediation technologies aimed at remediating chlorolignin waste polluting sites with consideration for environmental sustainability.

### 3.3 Chemical profile of sediment samples by GC–MS analysis

#### 3.3.1 Ethyl acetate extracts

**Figure 2:**
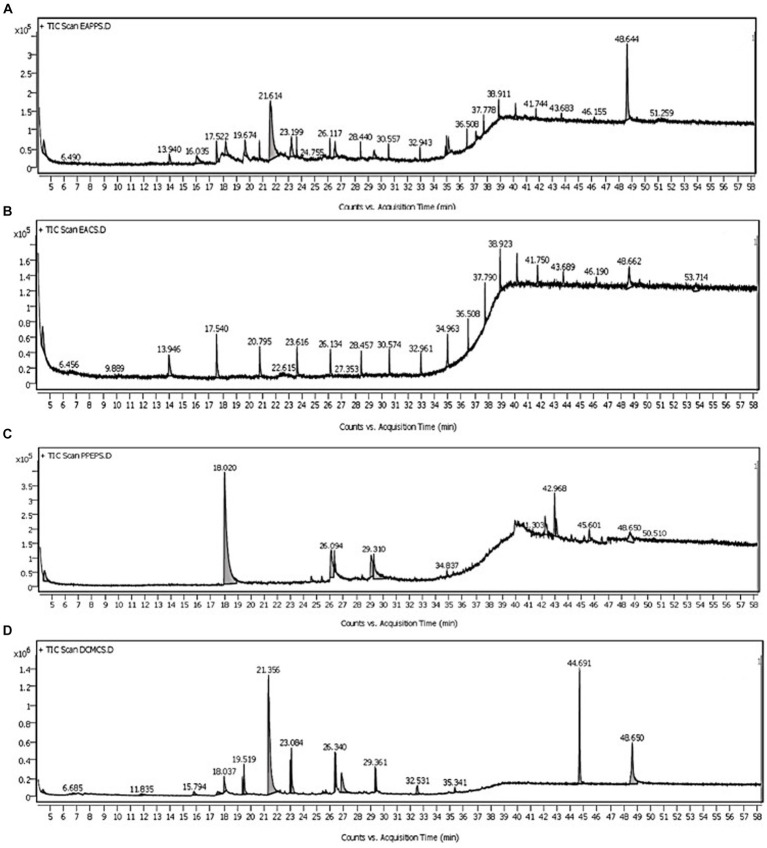
Representative total ion chromatogram by gas chromatography-mass spectrometry (GC–MS) analysis of organic pollutants extracted with different solvents from sediment samples PPS-1 and PPS-2 contaminated with paper mill wastewater **(A)** ethyl acetate; PPS-1 **(B)** ethyl acetate; PPS-2 **(C)** dichloromethane; PPS-1 **(D)** dicholoromethane; PPS-2.

In the GC–MS technique, the separation of plentiful organic compounds found in the extracts occurs based on retention times (RT), resulting in several minor and major peaks in the chromatogram, as shown in Figure 2. The organic compounds found in the samples have been identified as trimethylsilyl (TMS) derivatives by comparing their RT with the mass spectra of compounds available in the NIST library, and the closest matches are presented in Table 2. From Figure 2A, the major peaks detected in the ethyl acetate extract of PPS-1 at different RTs, such as 21.614 and 48.644 min, corresponded to trans-2,4-dimethylthiane, S,S-dioxide and furane 2,5-dimethyl, respectively. Furthermore, multiple minor peaks were observed at different RT values, and the respective compounds are presented in Table 2. In contract to PPS-1 extract, GC–MS analysis of PPS-2 extracted by ethyl acetate displayed predominant peaks at RT 13.940, 17.540, 20.795, 23.616, 28.457, 30.574, 32.961, 34.963, 36.508, 37.790, 38.923, 41.750, and 48.662 min (Figure 2B), which was identified as dodecamethy, tetradecamethyl; nonadecane, 2-methyl; furane 2,5-dimethyl; tetradecanoic methyl ester; dimethyl phthalate; hexadecanoic acid; benzene acetic acid; propanoic acid; benzoic acid; octadecane, 3-ethyl-5-(2-) and β-sitosterol, respectively. In addition to the mentioned peaks, very minor peaks were also detected at different RTs: 6.456, 9.889, 22.615, 27.353, 43.689, 46.190, and 53.174 min, as illustrated in Figure 2B. These minor peaks were, respectively, identified as spiro[2.4]heptane; 1-ethenyl-5-(1-propenylidene); 1-phenyl-1H-tetrazol-5-, trans-2,4-dimethylthiane, S,S-dioxide; benzene dicarboxylic acid; stigmasterol; heptacosane; and hexachlorobenzene. Tables 2, 3 represent a list of identified organic compounds detected at various RT in ethyl acetate extract of PPS-1 and PPS-2. The existence of these compounds in different sampling points underscores their persistence, as some of them do not undergo complete degradation during the secondary treatment process.

**Table 2:** Organic compounds identified in ethyl acetate extract of sediment sample (PPS-1) collected from Site-1, by GC–MS.

S. No.	RT	Height	Area %	Name of compounds
1	4.430	35,962	13.22	1-Cycloocten-5-yne, (Z)-
2	6.490	5,053	3.38	Benzyl (1,2,3-thiadiazol-4-y)carbamate
3	13.940	25,157	6.25	Cyclohexasiloxane, dodecamethyl
4	16.035	20,396	12.48	trans-2,4-Dimethylthiane, S,S-dioxide
5	17.522	56,849	9.83	Cycloheptasiloxane, tetradecamethyl
6	18.220	36,283	11.35	2,4-Di-tert-butylphenol
7	19.674	41,691	11.75	Dodecane, 1-fluoro-
8	20.332	6,792	1.53	n-Dodecylpyridinium chloride
9	20.772	47,941	6.00	Cyclooctasiloxane, hexadecamethyl
10	21.614	158,213	100.00	trans-2,4-Dimethylthiane, S,S-dioxide
11	23.199	52,854	17.09	1-Octanol, 2-butyl-
12	23.593	53,579	6.17	1,1,1,5,7,7,7-Heptamethyl-3,3-bis(trimethylsiloxy)tetrasiloxane
13	24.755	7,830	2.24	Cyclopentyl-methyl-phosphinic acid, 2-isopropyl-5-methyl-cyclohexyl ester
14	26.117	52,831	5.33	Octasiloxane, 1,1,3,3,5,5,7,7,9,9,11,11,13,13,15,15-hexadecamethyl
15	26.517	39,248	10.88	Isobutyl nonyl carbonate
16	27.049	7,310	2.29	trans-2,4-Dimethylthiane, S,S-dioxide
17	28.440	44,087	4.34	Nonylphenol
18	29.487	23,471	6.37	2-Azido-2,4,4,6,6-pentamethylheptane
19	30.557	42,561	4.62	Cyclononasiloxane, octadecamethyl
20	32.588	7,046	1.93	2-Azido-2,4,4,6,6-pentamethylheptane
21	32.943	34,766	4.02	Benzyl butyl phthalate
22	34.866	23,356	3.09	Cyclopentyl-methyl-phosphinic acid, 2-isopropyl-5-methyl-cyclohexyl ester
23	34.963	51,096	4.99	Dimethyl phthalate
24	35.106	42,169	9.05	4-n-Hexylthiane, S,S-dioxide
25	36.508	51,237	4.73	2,4, 6 Trichlorophenol
26	37.189	25,969	4.44	2-Methyl-4-keto-2-pentan-2-ol
27	37.778	51,957	4.39	4-((1-E)-3-Hydroxyl-1-propenyl)-2-methoxy
28	38.911	56,618	7.28	Octacosane
29	40.199	43,121	6.64	2-methoxy-4-vinylpheno
30	41.744	28,278	3.76	Pentadecanoic acid, ethyl ester
31	43.683	21,245	3.71	2-Propaneamine, 3-fluro-N-Phenyl
32	46.155	16,800	3.84	4-Chlorophenol
33	48.644	201,834	53.84	Furane 2,5-dimethyl
34	49.388	10,205	2.12	β-Sitosterol
35	51.259	9,812	2.19	Stigmasterol

RT, Retention time.

**Table 3:** Organic compounds identified in ethyl acetate extract of sediment sample, PPS-2, collected from Site-2, by GC–MS.

S. No.	RT	Height	Area %	Name of compounds
1	4.436	28,660	84.65	1-Cycloocten-5-yne, (Z)-
2	6.267	3,142	1.13	Undecane,3,5,-dimethyl
3	6.456	4,125	16.70	Spiro[2.4]heptane, 1-ethenyl-5-(1-propenylidene)
4	9.889	5,598	3.67	1-Phenyl-1H-tetrazol-5-
5	13.471	3,670	5.71	2,3-Heptadien-5-yne, 2,4-dimethyl
6	13.820	4,805	2.82	3-Hexanol,3-methyl
7	13.946	28,026	68.15	Dodecamethy
8	17.540	57,169	100.00	Tetradecamethyl
9	20.795	39,361	57.43	Nonadecane, 2-methyl
10	22.615	4,139	22.07	trans-2,4-Dimethylthiane, S,S-dioxide;
11	23.616	37,416	44.09	Pentadecanone
12	26.134	35,028	40.03	1-Decanol,2-hexyl
13	27.353	3,844	6.31	Benzene dicarboxilic acid
14	28.457	32,210	30.42	Furane 2,5-dimethyl
15	30.574	32,834	30.28	Tetradecanoic methyl ester
16	32.961	28,060	29.19	Dimethyl phthalate
17	34.963	42,944	49.91	Hexadecanoic acid
18	36.508	43,242	36.67	Benzene acetic acid
19	37.790	54,642	39.77	Propanoic acid
20	38.923	56,590	47.00	Benzoic acid
21	40.205	42,238	43.52	Phenol-2-methoxy-4-(1-propenyl)
22	41.750	28,209	38.19	Octadecane, 3-ethyl-5-(2-)
23	43.689	18,603	28.68	Stigmasterol
24	46.190	11,748	16.43	Heptacosane
25	48.662	27,937	95.04	β-Sitosterol
26	49.446	9,993	12.37	Hexachlorocyclohexane
27	53.714	11,611	56.79	Hexachlorobenzene

RT, Retention time.

#### 3.3.2 Dichloromethane extracts

The comparative GC–MS analysis of samples extracted with DCM from PPS-1 and PPS-2 showed the presence of a wide range of organic pollutants, as detailed in Tables 4, 5, respectively. The GC–MS chromatogram of PPS-1 revealed few dominant peaks at RT 18.020 and 42.968 min (Figure 2C), which were identified using the NIST library as adamantane, 1-isothiocyanato-3-methyl-; and arsenous acid, tris(TMS) ester, respectively. Moreover, the total ion chromatogram (TIC) of PPS-1 showed several minor peaks at RT 26.094, 34.837, 41.303, 45.601, 48.650, and 50.510 min (Figure 2C). These peaks were characterized as dodecanoic acid; 3-methylpyrazolobis(diethylboryl)hydroxide; tris(tert-butyldimethylsilyloxy)arsane; 9,12-octadecadienoic acid(z,z)-2,3-dihydroxypropyl ester; 2 ethyl 4–6 dimethyl-1,3,5-trixane; and ethane, 1,1-diethoxy. Furthermore, some minor peaks were observed at different RT values, and the compounds corresponding to these minor peaks are provided in Table 4. In contrast to the previously mentioned observations, the GC–MS chromatogram of dichloromethane-extracted samples, collected from PPS-2, revealed multiple major and minor peaks at various RT, as illustrated in Figure 2D. These peaks were found to correspond to different organic compounds, and their identities are provided in Table 5. The major detected peaks at RT of 21.356, 44.691, and 48.650 min, which were identified as octadecane, β-sitosterol, and 1-decanol, 2-hexyl, respectively. Moreover, there were additional minor peaks observed at RT of 6.685, 11.835, 18.037, 19.519, 23.084, 26.340, 29.361, 32.531, and 35.341 min, as depicted in Figure 2D. This peak corresponded to 2-azido-2,4,4,6,6-pentamethylheptane; pentadecanoic acid, ethyl ester; dimethyl phthalate; 1,3-dioxane,2-methy; pentadecanoic acid; furane 2,5-dimethyl; 2,4-di-tert-butylphenol; dodecamethy; and propanoic acid. Furthermore, various minor peaks were observed at different RT, as indicated in Figure 2D. However, these particular peaks could not be identified since their corresponding compounds were not present in the NIST library. In our study, a substantial number of identified compounds were detected in the ethyl acetate extract of both sediment samples, as summarized in Tables 2 3.

**Table 4:** Organic compounds identified in dichloromethane extract of sediment sample, PPS- 1 collected from Site-1, by GC–MS.

S. No.	RT	Height	Area %	Area Sum%	Name of compounds
1	4.419	34,969	7.26	3.27	1-Cycloocten-5-yne
2	18.020	390,232	100.00	45.04	2,4-Di-tert-butylphenol;
3	18.020	19,248	1.92	0.86	Adamantane, 1-isothiocyanato-3-methyl-;
4	25.379	19,436	1.65	0.74	(trans-2,4-Dimethylthiane, S,S-dioxid)
5	26.094	94,454	18.78	8.46	Dodecanoic acid;
6	26.334	72,486	3.67	1.65	(trans-2-methyl-4-n-pentylthiane, S,S-dioxide)
7	26.415	25,218	1.24	0.56	2-Azido-2,4,4,6,6,8,8-heptamethylnonane
8	28.412	15,757	1.11	0.50	trans-2,4-Dimethylthiane, S,S-dioxide
9	29.115	76,984	13.36	6.02	Octadecanoic acid;
10	29.310	88,463	20.39	9.18	Hexadecanoic acid
11	34.837	23,382	1.45	0.65	3-Methylpyrazolobis(diethylboryl)hydroxide;
12	41.303	20,325	2.10	0.95	Tris(tert-butyldimethylsilyloxy)arsane;
13	42.253	64,161	8.04	3.62	Pentatriacontane
14	42.968	151,140	11.12	5.01	Arsenous acid, tris(trimethylsilyl) ester;
15	43.089	63,425	6.59	2.97	2-methoxy phenol
16	44.227	21,207	1.56	0.70	Arsenous acid, tris(trimethylsilyl) ester;
17	45.223	14,451	1.54	0.69	Ethane,1,1-diethoxy
18	45.601	39,870	3.89	1.75	9,12-octadecadienoic acid(z,z)-2,3-dihydroxypropyl ester
19	46.528	22,496	2.01	0.90	cis-9-Hexadecenoic acid
20	48.650	33,372	11.10	5.00	2 ethyl 4–6 dimethyl-1,3,5-trixane
21	50.510	13,804	3.25	1.46	Ethane, 1,1-diethoxy

RT, Retention time.

**Table 5:** Organic pollutants identified in dichloromethane extract of sediment sample, PPS-2 collected from Site-2, by GC–MS.

S. No.	RT	Height	Area %	Name of compounds
1	4.436	32,020	2.52	1-Cycloocten-5-yne, (Z)-
2	6.685	15,144	1.24	2-Azido-2,4,4,6,6-pentamethylheptane
3	11.835	16,228	2.73	Pentadecanoic acid, ethyl ester
4	15.794	40,186	3.17	2-methoxy phenol
5	17.534	33,648	3.51	Hexadecanoic acid
6	18.037	173,199	7.36	Dimethyl phthalate
7	19.411	188,351	5.05	trans-2-Methyl-4-n-butylthiane
8	19.519	334,953	11.18	1,3-Dioxane,2-methy
9	21.356	1,313,818	100.00	Octadecane
10	22.987	360,118	9.29	Hexachlorobenzene
11	23.084	499,235	17.03	Pentadecanoic acid
12	25.430	28,511	1.36	Tritetracontane
13	25.653	38,619	1.20	Hexachlorobenzene
14	26.340	434,396	8.87	Furane 2,5-dimethyl
15	26.409	336,889	7.98	Octadecanoic acid
16	26.838	212,391	19.16	Isobutyl nonyl carbonate
17	29.361	273,514	5.59	2,4-Di-tert-butylphenol
18	29.413	231,326	6.27	Heptacosane
19	32.531	97,032	5.79	dodecamethy
20	35.341	55,199	2.19	Propanoic acid
21	44.691	1,272,313	41.47	β-Sitosterol
22	48.650	449,803	33.85	1-Decanol,2-hexyl

RT, Retention time.

### 3.4 Bacterial communities profiling by high throughput sequencing

#### 3.4.1 Isolation of metagenomic DNA and PCR amplification

The DNA samples recovered from PPS-1 and PPS-2 using a soil DNA isolation kit were of high quality. The A_260/230_ ratio for both PPS-1 and PPS-2 fell within the range of 1.89 to 1.88, while the A_260/280_ ratio was between 2.07 and 2.10, respectively. The concentration of the metagenomic DNA isolated from the sample was 233.3 and 261.8 ng/μl (260 nm). A summary of the quality and quantity of the extracted metagenome has been presented in Supplementary Table S2.

#### 3.4.2 Quality and quantity of MiSeq library

The quality check amplified 2× 300 MiSeq library analyzed on the 4,200 Tap Station system showed three peaks; minimum, optimum, and upper peaks as shown in Supplementary Figure S2. The results of the analyzed library are depicted in Table 6A.

**Table 6:** (A) A summary of quality and quantity of MiSeq library checked on 4,200 Tape Station System; (B) Metagenome high throughput sequence read count statistics.

A. A summary of quality and quantity of MiSeq library checked on 4,200 Tape Station System						
Sample	From (bp)	To (bp)	Average size (bp)	Conc. (ng/μl)	Region molarity (nmol/l)	% of total
PPS-1	494	797	621	10.8	27.0	75.70
PPS-2	497	837	628	9.74	24.2	69.52

B. Metagenome high throughput sequence read count statistics						
Sample	Raw reads	Raw total bases	Raw data (in MB)	HQ Reads	HQ total bases	HQ data (in MB)
PPS-1	1,53,675	92,268,970	92.27	1,15,665	56,444,791	56.44
PPS-2	1,62,691	97,697,551	97.70	1,19,386	57,992,884	57.99

MB, Megha base; HQ, High quality; Conc, Concentration; %, Percent; bp, Base pair.

#### 3.4.3 Metagenomics sequencing reads

In the present study, HTS of the V3 and V4 region of the 16S rRNA gene derived from PPS1 and PPS2, a total of 1,53,675 and 1,62,691 raw sequence reads were generated, respectively. After trimming low-quality reads, the MiSeq sequencing yielded 115,665 and 119,386 high-quality paired reads with an average length of 250 bases in PPS-1 and PPS-2, respectively which were further used for *de novo* assembly. Table 6B provides a summary of sequence reads that have successfully passed through each filter. Moreover, a total of 2,594 OTU was predicted across all the samples, utilizing the Greengene database. OTU cluster analysis revealed a total of 1,249 OTUs were identified after clustering at a 97% similarity level from 1,15,665 high-quality reads of PPS-1. A total of 1,345 OTU was obtained from unique high-quality read sequences of PPS-2 sample. The average OTUs of the contaminated PPS-2 samples were higher than those of the PPS-1 samples (Supplementary Table S3). A total of 56,444,791 and 57,992,884 were derived from high-quality total base reads of PPS-1 and PPS-2, respectively. The metagenome from sample PPS-1 gave 56.44 High Quality (HQ) data (In MB) reads, while in the sample of PPS-2 were 57.99 HQ data (In MB) reads (Table 6B).

#### 3.4.4 Diversity analysis for bacterial communities

##### 3.4.4.1 Abundance of OTU

**Figure 3:**
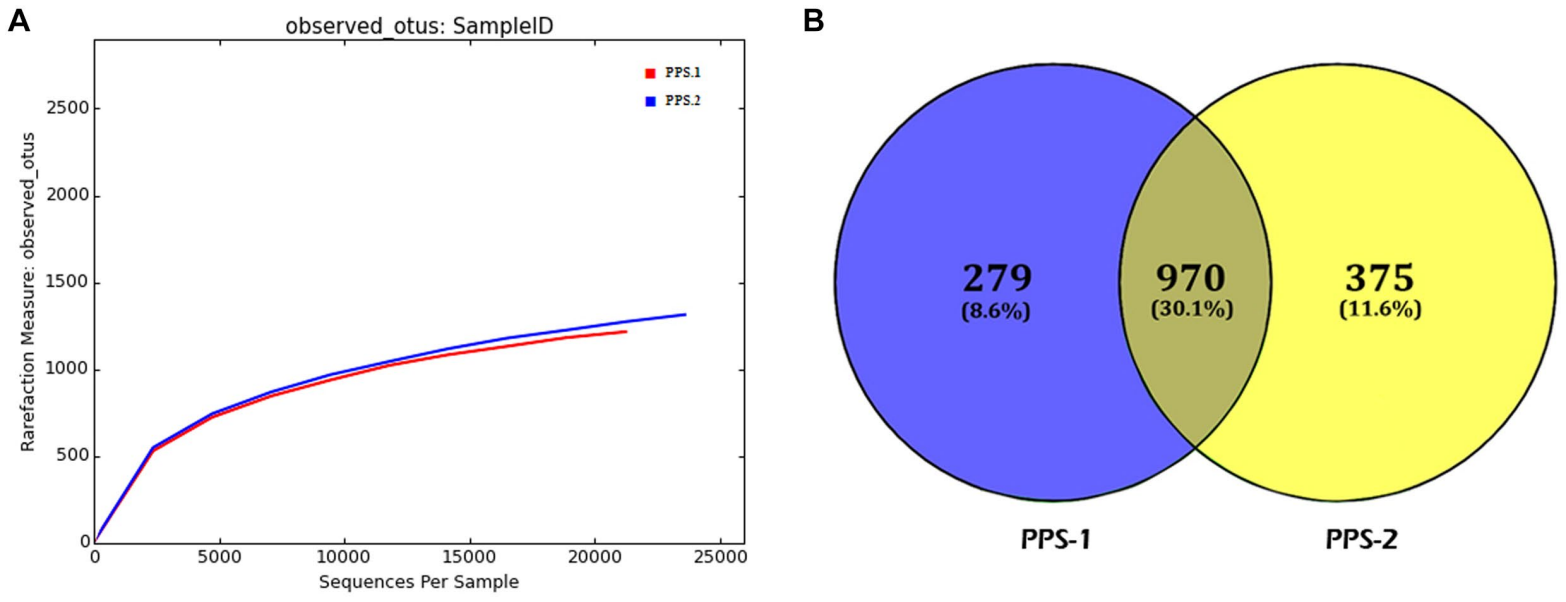
Bacterial communities diversity analysis in sediment samples. **(A)** Rarefaction curve showing bacterial communities diversity over the number of reads of the sequences (OTU) recovered from polluted sediment samples. The horizontal axis represents the number of sequences, while the vertical axis illustrates the diversity of the community. The sediment sample PPS-2 had the highest bacterial diversity compared to the PPS-1. **(B)** Venn diagram of samples shown the overlap of the bacterial communities from sediment samples based on OTU (0.97 similarity).

The rarefaction curves illustrated that the abundance of OTUs varied between PPS-1 and PPS-2, as depicted in Figure 3A. Based on the data, it appears that PPS-2 displayed a higher bacterial diversity (richness) compared to the samples from PPS-1. Notably, both the rarefaction curves (Figure 3A) for observed OTU approached near-saturation. Additionally, a Venn diagram was developed to illustrate the overlaps and distinctions in the bacterial communities within the sediment samples. This analysis was based on the proportion of unique and shared OTUs identified among the sediment samples, as shown in Figure 3B. The diagram vividly illustrates the variations in the number of bacterial communities among the different samples. In this study, notable differences were observed in the number of bacterial OTUs between PPS-1 and PPS-2. Specifically, 1,249 OTUs were identified in PPS-1, while PPS-2 exhibited 1,345 OTUs based on Illumina analysis. In both samples, there were unique OTUs, and these unique OTUs accounted for 8.6 to 11.6% of the total sequences. Specifically, the number of unique OTUs in PPS-1 and PPS-2 was 279 and 375, respectively. The total number of OTUs shared between the two sediment samples was 970, representing 30.1% of the total number of OTUs observed, which amounted to 2,594. When the same OTUs are present in two distinct samples, it signifies the presence of the same bacterial species in different sediment samples throughout the study.

##### 3.4.4.2 Variation in alpha diversity indices

To gain a deeper understanding of bacterial diversity within the samples, α-diversity indices, specifically the Shannon and Simpson indices, were computed based on the number of retrieved OTUs (Feranchuk et al., 2018; Walters and Martiny, 2020). The results from the Shannon and Simpson indices underscore that the sediment PPS-1 had the low community richness (Shannon index: 7.99; Simpson index: 0.987), whereas the PPS-2 sample had the highest community richness (Shannon index: 8.23; Simpson index: 0.991). The details of the observed OUT and computed Shannon and Simpson are listed in Supplementary Table S3.

##### 3.4.4.3 Taxonomic profiles of the bacterial community

**Figure 4:**
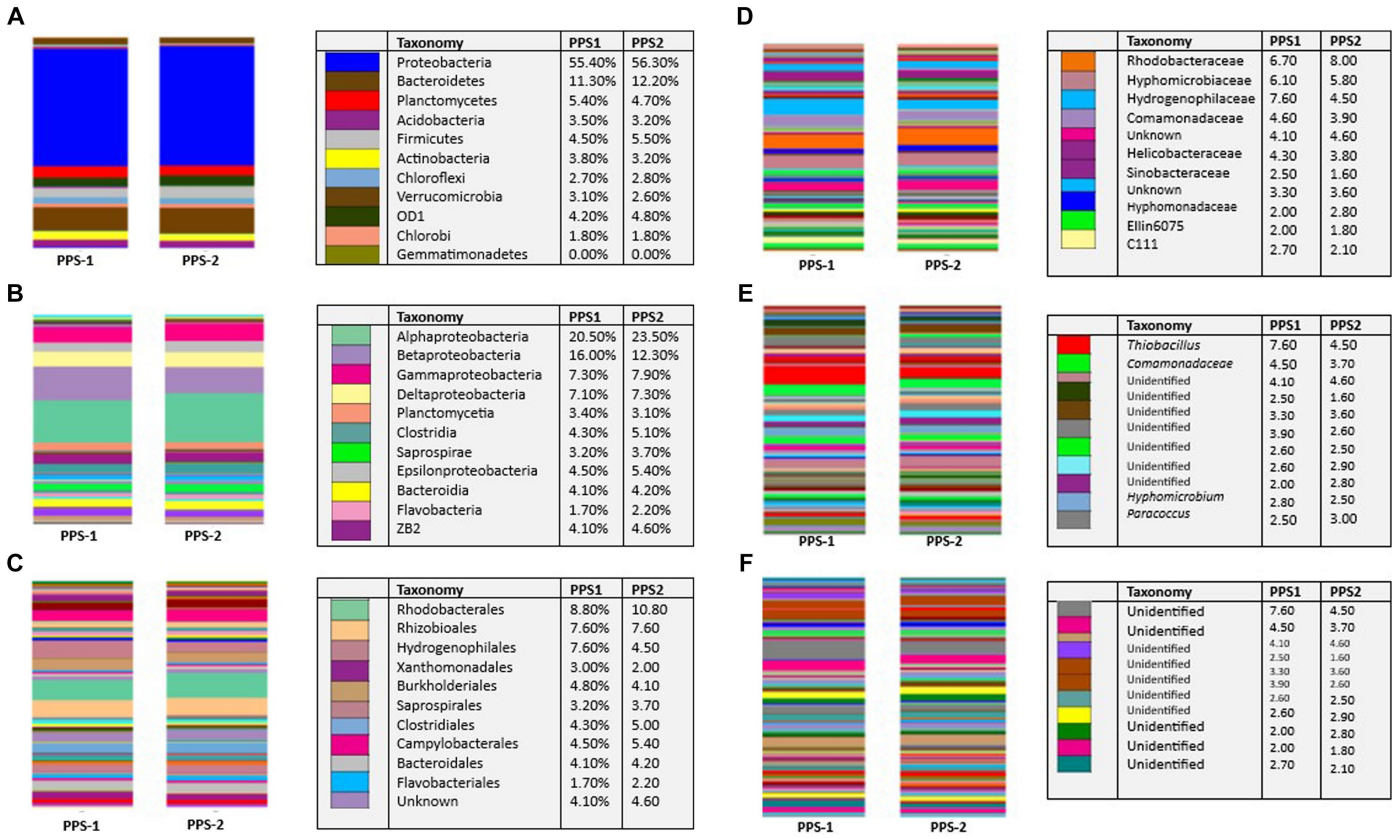
Comparison of bacterial community structure in PPS-1 and PPS-2 samples at different levels. Stacked bar chart of relative abundance (Bray-Cutis distance) of most dominant bacterial communities within the samples at different level of **(A)** Phyla **(B)** classes **(C)** order **(D)** family **(E)** genera **(F)** species. The relative abundance is expressed as a percentage of the total effective bacterial sequences in various samples, classified using the RDP Classifier. Low abundant phyla shown as “Unknown.” “Unclassified” signifies sequences which did not match any known sequence in the database.

According to the annotation process, a total of 2,594 OTUs were identified in both sediment samples. These OTUs were further classified into different taxonomic levels, as depicted in Figure 4. Illumina sequencing analysis identified 1,249 OTUs (from 1,15,665 high-quality reads) recovered from PPS-1 that were taxonomically classified to different phyla, classes, order, families, genera, and species. In this study, the RDP Classifier was utilized to assign the effective bacterial sequences to different phylogenetic taxa. Notably, bacterial communities found in sediment samples exhibited significant differences at each taxonomic unit level, as illustrated in Figure 4.

The phylum-level bacterial diversity was analyzed through the HTS of hypervariable segments of the 16S rRNA gene derived from PPS-1 and PPS-2 samples. A total of 37 distinct phyla were detected in PPS-1 (Supplementary Table S4) of which 23 phyla have a known cultivable representative like *Acidobacteria, Actinobacteria, Chloroflexi, Bacteroidetes, Chloribi, Chloroflexi*, and *Proteobacteria*; whereas 13 phyla do not have cultivable representative so far, such as OD1, TM7, NKB 19, WPS-12, WS6, SR1, NKB19, WS3, GN02, FCPU426, TM6, WWE1, and OP11 (Supplementary Table S4). One bacterial phylum was not assigned any phyla. In addition, 48 classes, 75 orders, 136 families, 132 genera, and 31 species representing cultivable bacteria were identified in PPS-1 (Supplementary Table S4). In contrast to PPS-1, a total of 35 distinct phyla were detected in PPS-2, of which 20 phyla have a known cultivable representative, including *Acidobacteria, Actinobacteria, Bacteroidetes, Chloroflexi, Chloribi, Chloroflexi, Proteobacteria, Spirochaetes, Nitrospirare, Verurucomicrobia, Firmicutes*, and *Planctomycetes*; whereas 15 phyla do not have cultivable representative so far, such as OD1, TM7, WPS-2, NKB19, GN04, SR1, WS3, TM6, OP11, GN02, BRC1, WWE1, and FCPU426. Moreover, 45 classes, 75 orders, 138 families, 152 genera, which represent 48 cultivable species were detected in PPS-2 (Supplementary Table S5). At the phylum level, the relative abundance analysis indicated that *Proteobacteria* was the main dominant group (Figure 4A), accounting for 55.40 and 56.30% within PPS-1 and PPS-2, respectively. Moreover, the second most abundant phylum was *Bacteroidetes*, accounting for 11.30 and 12.20% in PPS-1 and PPS-2, respectively. Additional low-abundant phyla were also represented by *Planctomycetes, Acidobacteria, Firmicutes, Actinobacteria, Chloroflexi, Verrucomicrobia*, OD1, and *Chlorobi* as shown in Figure 4A.

At the class level, a total of 37 distinct classes were detected in PPS-1. *Alphaproteobacteria, Betaproteobacteria, Gammaproteobacteria, Deltaproteobacteria* were major bacterial communities in both PPS-1 and PPS-2, with a relative abundance of 20.15–23.50%, 16.00–12.30%, and 7.30–7.90%, respectively. Other low abundant classes included *Gammaproteobacteria, Deltaproteobacteria, Planctomycetia, Clostridia, Saprospirae, Epsilonproteobacteria*, and *Bacteroidia* (Figure 4B).

At the order level, Rhodobacterales was the dominant group in sediment samples, accounting for 8.80 and 10.80% in PPS-1 and PPS-2, respectively. The other most abundant orders in PPS-1 and PPS-2 were Rhizobioales (7.60 and 7.60%), Hydrogenophilales (7.60 and 4.50%), while the other low abundant classes order included Burkholderiales (4.80 and 4.10%), Saprospirales (3.20% and 3.70), Clostridiales (4.30% and 5.00), Campylobacterales (4.50 and 5.40%), Bacteroidales (4.10–4.20%), and Flavobacteriales (1.70–2.20%) (Figure 4C).

At the family level, the relative abundance analysis indicated that Hydrogenophilaceae was the main dominant family in PPS-1, accounting for 7.60% (Figure 4D). The second most abundant family was Rhodobacteraceae (6.70%) in PPS-2. In contracts to PPS-1, the most abundant family in PPS-2 was Rhodobacteraceae, with relative abundance of 8.00%. Other less abundant families in PPS-1 and PPS-2 included Hyphomicrobiaceae, Comamonadaceae, Helicobacteraceae, Hyphomonadaceae. A substantial portion of the bacteria could not be identified beyond the Order and family level, with the majority categorized as “Unknown.”

At the genus level, the major genus in PPS-1 and PPS-2 was *Thiobacillus* (7.60 and 4.50%) (Figure 4E). As depicted in Figure 4E, a significant number of bacteria could not be identified at the genus level; labeled as “Unidentified,” and their relative abundances are displayed in Figure 4E.

At the species level, all the detected bacterial genera are unassigned to any species. As shown in Figure 4F, a significant portion of the bacteria could not be identified at the genus level, with the majority being labeled as “Unidentified,” and their relative abundances are visually presented in Figure 4F. The summary of assigned bacteria at different taxonomic level are given in Table 7 and Supplementary Table S6.

**Table 7:** Dominant and specific bacterial taxa identified in sediments samples at different taxonomic levels.

Sample	PPS-1	PPS-2
Hypervariable region	V3-V4	V3-V4
Sequencing platform	Illumina MiSeq	Illumina MiSeq
Phylum	37	35
Class	48	45
Order	75	75
Family	136	138
Genus	132	152
Species	31	48

**Figure 5:**
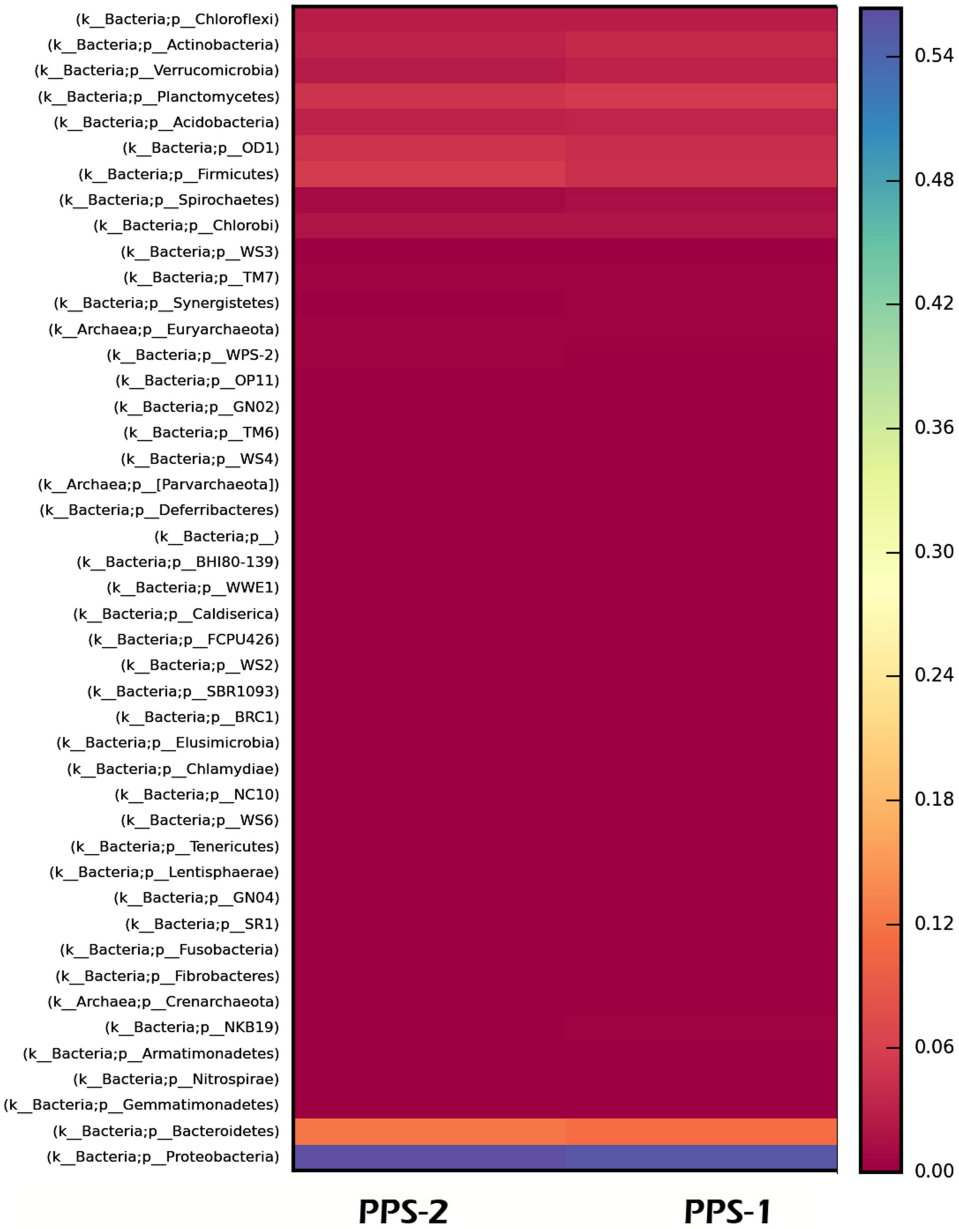
Heat map showing relative abundance of major bacterial phyla identified by metagenomic sequencing in the two metagenomic libraries prepared from two metagenome obtained from sediment sample, PPS-1 and PPS-2, collected from different locations. The heatmap legend displays the percentage abundance of phyla in each of the samples.

**Figure 6:**
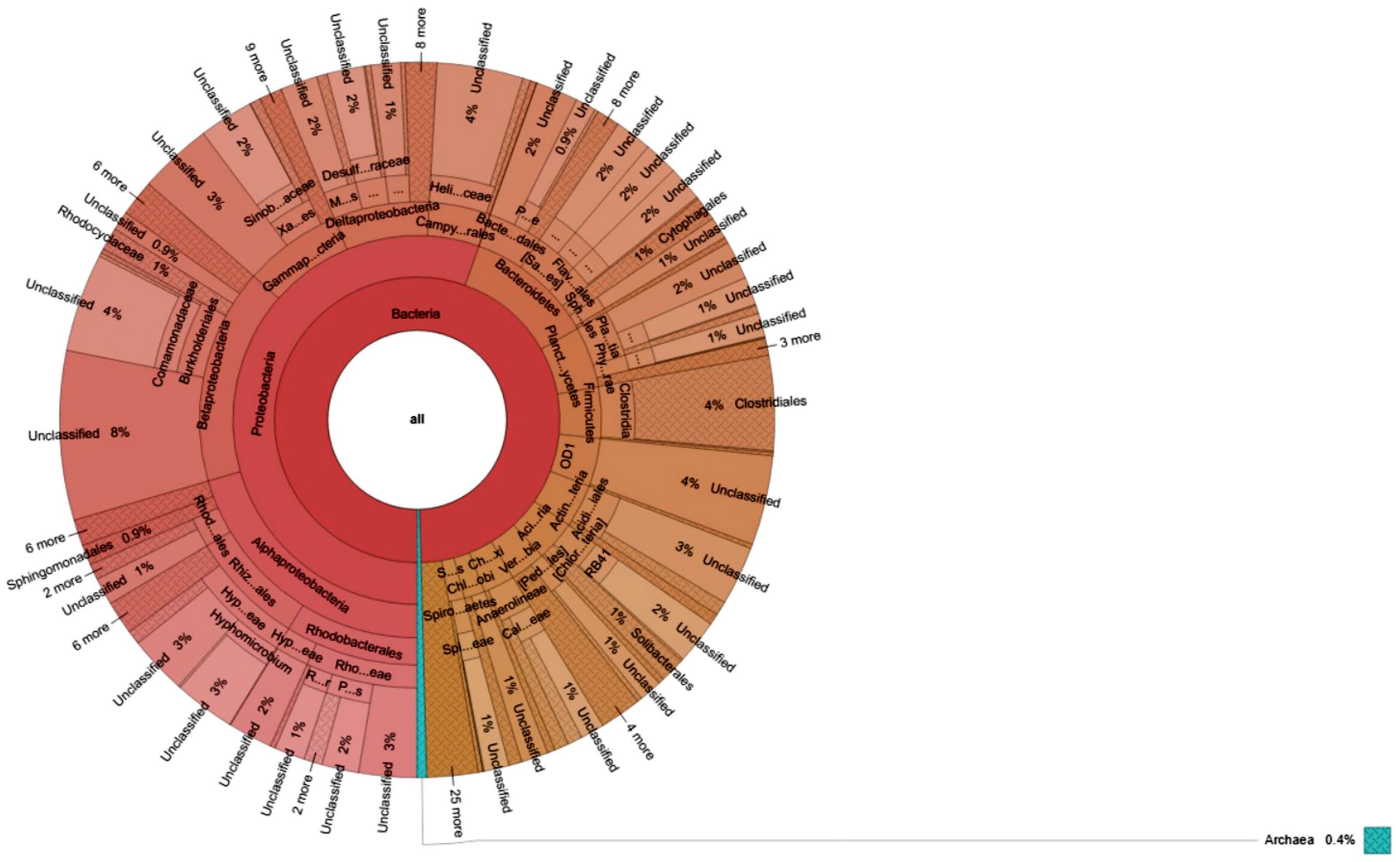
Krona charts show the taxonomic composition of microbial communities grown in sediment sample, PPS-1. The outermost to inner circles represent species, genera, family, orders, and phyla levels, respectively. Percent (%) numbers are indicated the bacterial abundance.

**Figure 7:**
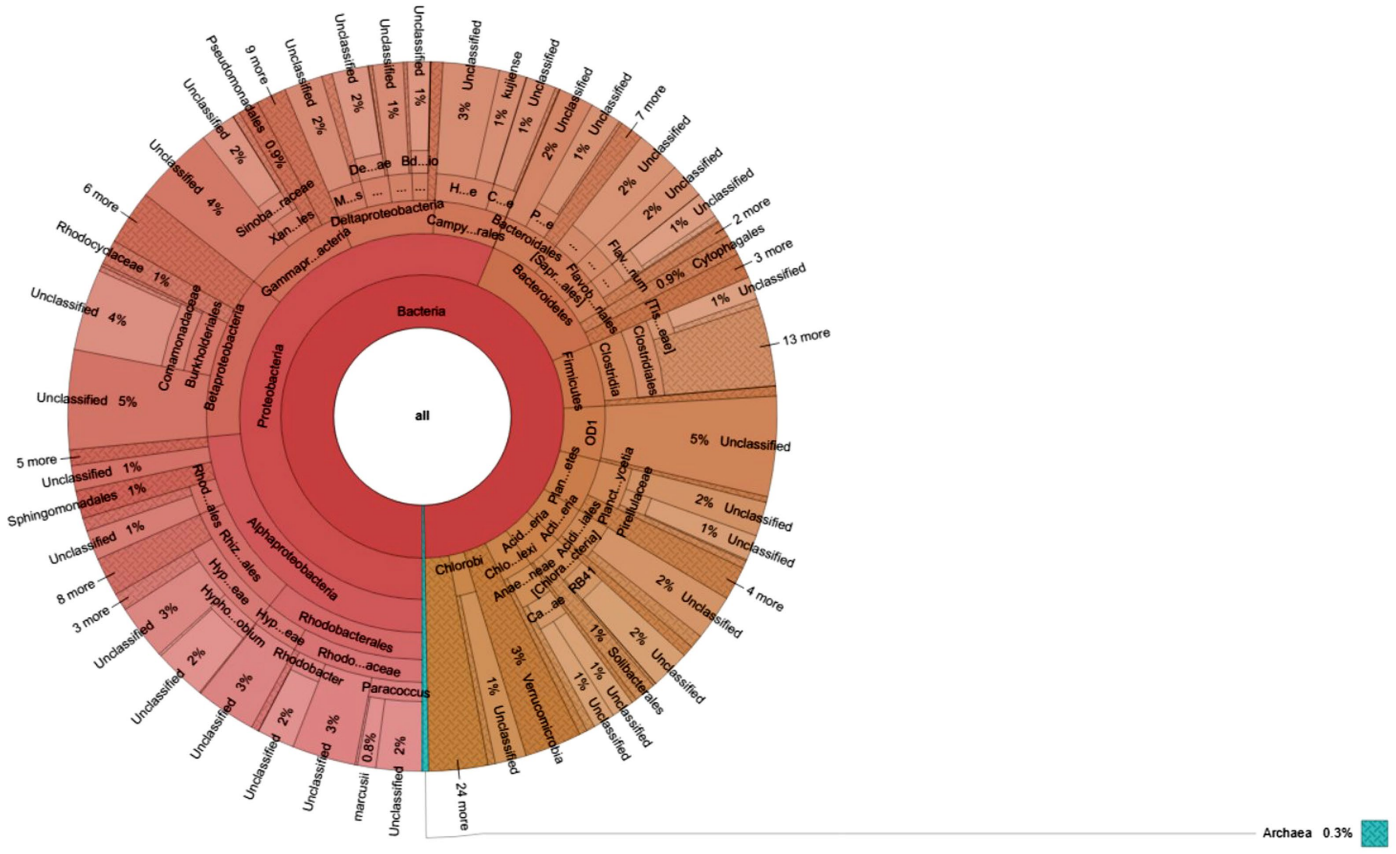
Krona charts show the taxonomic composition of microbial communities grown in sediment sample, PPS-2. The outermost to inner circles represent species, genera, family, order, and phyla levels, respectively. Percent (%) numbers are indicated the bacterial abundance.

Furthermore, a heatmap illustrating the most abundant OTUs revealed distinctions between the sediment samples from Site-1 and Site-2 (Figure 5). The heatmap grouped the sediment samples but also separated them into distinct clusters based on the correlation between the most abundant OTUs (Figure 5). Krona charts (Figures 6, 7), known for their visually striking presentation, depict the relative abundance of taxa at various hierarchical levels within the sediment samples.

## 4 Discussion

### 4.1 Variation in physico-chemical parameters

PPMs extensively utilize huge volumes of freshwater in several processing steps, including cleaning, chemical pulping, bleaching, and paper development, simultaneously generating vast volume of effluent as a by-product (Kumar A. et al., 2021; Conte et al., 2022). The generated effluent is heavily loaded with a multifaceted blend of organic, inorganic, and organometallic pollutants like lignins, tannins, resins, and their derivatives, fatty acids, sulfur compounds, including chloroorganics like lignins, phenols, hydrocarbon, dioxins, furans, and resin acids (Castro et al., 2018; Kumar and Verma, 2023). Improper discharge of effluent can have serious impacts on the environment, leading to contamination of natural resources and posing serious health risks (Ratia et al., 2012; Orrego et al., 2019; Haq et al., 2022). In this study, two sites were selected for the collection of sediment samples. Notably, significant differences in the physico-chemical properties were detected between PPS-1 (Site-1) and PPS-2 (Site-2), as shown in Table 1. In our study, the pH of both sediment samples were found to be alkaline. The elevated pH in the sediment can likely be attributed to the residues of sodium hydroxide and sodium sulfide, commonly used in the pulping process, as well as the high concentrations of ions such as K^+^, Na^+^, and Cl^−^. Furthermore, high EC was found in sediment samples. EC serves as a valuable indicator of soil health, representing overall salinity. The elevated EC values are likely associated with the presence of hydroxides, carbonates, bicarbonates, and salts, along with ions such as K^+^, Na^+^, and Cl^−^, commonly used in the pulping and bleaching processes. The presence of salt and Cl^−^ may result from byproduct reactions involving sodium sulfide and/or sodium hydroxide during the pulping process. Consequently, the salinity of the soil/sediment’s is unsuitable for agricultural purposes, as high salt levels can adversely impact crop yields, nutrient availability, and the activity of the microbial community inhabiting the soil. It’s worth noting that several authors have reported elevated levels of these cations and anions in wastewater (Eskelinen et al., 2010; Hajdu-Rahkama and Puhakka, 2022) and paper sludge (Chandra et al., 2017; Singh et al., 2020). In India, farmers widely use chloride-containing bleach wastewater in irrigation as it not adsorbed by soil and moves quickly travels with soil water, gets adsorbed by crops, and ultimately accumulates in the leaves. Chloride is typically considered more toxic to flora and fauna, including the microbial community, compared to sulfates. It was observed that the organic carbon was found in high concentration in PPS-1 and PPS-2. The elevated organic matter content is attributed to the presence of residual chlorolignin fiber material that persists even after undergoing secondary treatment. This observation aligns with findings previously reported by other researchers who reported the high load of various physico-chemical pollutants in discharged paper sludge and sediment (Chandra et al., 2017; Singh et al., 2020). In this study, the chlorophenol, lignin, and total phenol concentrations of PPS-2 were lower than those in PPS1, as represented in Table 1. The presence of chlorophenols is attributed to higher molecular weight compounds generated as degradation product of lignin and substantial bleaching of pulp by chemical agents. The phenol content of sediment sample PPS-1 was recorded as 351 mg kg^−1^, which was higher compared to PPS-2. Chlorophenols, a common group of toxic pollutants from pulp manufacturing, pose a significant risk to soil, water, and aquatic ecosystems in the food chain, endangering humans and other organisms (Hoovield et al., 1998). The high chlorophenol content indicates that chlorophenol has a toxic impact on the indigenous soil microbial community. The results of our study found strong support in previous observations made by various researchers who reported lignin, chlorophenol, and total phenol in PPMW (Kumar and Chandra, 2021; Sharma et al., 2021a) and activated sludge (Sharma et al., 2023). This study confirmed that the sediment sample is highly contaminated with the inorganic and organic load of various pollutants discharged in PPMW even after the secondary treatment process. We believe that the biological and chemical profiles of soils in proximity to these informal dumps persistently experience substantial impacts.

### 4.2 Heavy metals content in sediments

The elevated levels of heavy metals pose a severe threat to the environment and humans health hazards due to their non-destructive nature, tending to bioaccumulate and biomagnify in the food chain. In our study, Al, Fe, and Ti, showed to be found in high concentration in the sediment samples. The presence of Fe might be result from the corrosion of iron vessels and pipes during the alkaline digestion process used in pulping wood chips (Chandra et al., 2017). Our results validated the earlier findings, where excess concentration of metals was found in pulp paper mill waste (An et al., 2021; Haq et al., 2022). Yadav and Chandra (2018) examined the physico-chemical characteristics of sediment samples collected from the pulp-paper mill waste dumping site and reported the high concentration of heavy metals Zn, Fe, Cd, Cr, Mn, Ni, and Cu. Elevated concentrations of these metals can impact soil permeability, texture, and, over time, lead to a reduction in the soil microbial community, ultimately soil productivity (Zhang et al., 2016; Feng et al., 2018; Zhu et al., 2023). Heavy metals like Cr and lead (Pb), along with various phenolic compounds, are categorized as “priority pollutants” by the USEPA (United States Environmental Protection Agency). These substances are known to have significant mutagenic and carcinogenic effects on both animals and humans, posing a serious health risk. The significantly elevated values of the physico-chemical parameters observed in PPS-1 and PPS-2 suggest a substantial pollution load due to discharged wastewater. The exceptionally high levels of these pollution parameters indicate the ecotoxicological and hazardous nature of the sediment.

### 4.3 Identified organic pollutants by GC–MS

The GC–MS is a robust analytical tool extensively employed to identify and quantify organic compounds in a complex environmental samples (Špánik and Machyňáková, 2018; Maurin et al., 2023). In the present study, the GC–MS technique has been utilized to detect and characterize organic compounds in the organic extract, extracted with two different solvents namely ethyl acetate and DCM, derived from PPS-1 and PPS-2. The GC–MS analysis of ethyl acetate extracts from PPS-1 identified over 35 organic compounds including some that are known mutagens, carcinogens, and environmental endocrine disruptors. Pentadecanone, 1-decanol,2-hexyl, benzene dicarboxilic acid, tetradecanoic methyl ester, octadecane, 3-ethyl-5-(2-), heptacosane, and β-sitosterol, constitute the primary components found in the wastewater remaining after secondary treatment of wastewater. Phenolic compound and phthalates are documented as potential EDCs. Stigmasterol and β-sitosterol, prevalent phytosterols extracted from wood during pulp development processes (Cook et al., 1997; Christianson-Heiska et al., 2008), have the potential to end up in the final discharge in pulp mill effluents. β-Sitosterol is known to induce various endocrine effects in fish, including estrogenic activity such as plasma vitellogenin (VTG) induction (Mellanen et al., 1996; Tremblay and Van Der Kraak, 1998; Orrego et al., 2009), as well as alterations in plasma sex hormone levels and changes in gonadal steroidogenesis (MacLatchy and Van Der Kraak, 1995 Orrego et al., 2010). These chemical pollutants were previously reported to be present in activated sludge (Tripathi et al., 2022), sludge (Chandra et al., 2017), and untreated or treated PPMW (Balabanič et al., 2021 Sharma et al., 2021b). In our study, the GC–MS analysis of ethyl acetal extracted samples from PPS-2 revealed the presence of pentadecanone, 1-decanol,2-hexyl, benzene dicarboxilic acid, tetradecanoic methyl ester, dimethyl phthalate, hexadecanoic acid, propanoic acid, benzoic acid, octadecane, 3-ethyl-5-(2-), stigmasterol, heptacosane, β-sitosterol, etc. The existence of these compounds is also corroborated with the previous observations (Chandra et al., 2017; Liang et al., 2021; Tripathi et al., 2022). The identified compounds may pose a significant threat to aquatic organisms due to their cytotoxic, genotoxic, and carcinogenic properties, as recognized the potential EDCs by USEPA (2012).

GC–MS analysis of DCM extracted samples from PPS-1 and PPS-2 revealed the presence of several organic compounds. The presence of organic contaminants suggests that the sediments pose an environmental risk to aquatic flora and fauna at waste disposal sites. Similar compounds have been reported by various researchers (Chandra et al., 2017; Tripathi et al., 2022). Notably, the detected compounds like hexadecanoic acid and octadecanoic acid have been reported as potential EDCs by the USEPA (2012). Octadecanoic acid and hexadecenoic have been detected and identified in the final discharge of PPMs by earlier researchers (Yadav and Chandra, 2018; Kumar and Chandra, 2021; Sharma et al., 2021a).

In this study, several other organic compounds in the ethyl acetate and DCM extract of PPS-1and PPS-2 have also been detected. These might be generated as a residual pollutant of the pulping and bleaching process during pulp production. Moreover, these compounds were likely produced during microbial biotransformation in the course of effluent treatment, potentially leading to the formation of sulfurous compounds. Nevertheless, the role of numerous other organic contaminants detected in the sediment remains a subject of interest and warrants further study for the sake of environmental safety. This finding underscore that ethyl acetate serves as an excellent organic solvent for extracting pollutants from the waste discharged from PPMs, even after secondary treatment. These conclusions are well supported by earlier results reported by Yadav and Chandra (2018).

### 4.4 Quality and quantity of isolated DNA

The sediment samples under the current experiment were rich in microorganisms as well as organic matter especially humic acids which denature DNA by binding phenolic groups to amides. The ratio of the readings at 260 nm and 230 nm (A_260_/A_230_) provides an estimate of DNA purity with respect to contaminants that absorb UV light. A A_260_/A_230_ ratio less than 2 indicates humic acid contamination (Ning et al., 2009), while A_260_/A_280_ ratio of less than 1.8 indicates protein contamination (Maniatis et al., 1982). Samples with a A_260_/_230_ ratio between ~2.0–2.2 and 260/280 ~ 1.7–2.0 are assumed as “pure” (Demkina et al., 2023). In our study the A_260_/_230_ ratio for PPS-1 and PPS-2 was in range of between 1.89 and 1.88 demonstrates the lack of organic impurities (Olson and Morrow, 2012). The ratio of absorbance at 260 nm and 280 nm is used to assess the purity of DNA. In general, the extracted DNA is free from inhibitors. In this study, the A_260_/_280_ ratio was between 2.07 and 2.10, for PPS-1 and PPS-2, respectively.

The maximum yield of DNA was obtained in the sediment collected from Site-I. The quantity of DNA amounted to around 10.8 ng/μl and and 9.74 ng/μl isolated from PPS-1 and PPS-2, respectively.

### 4.5 Metagenomics analysis

#### 4.5.1 High throughput sequencing reads data

Chlorolignin contamination of soil, sediment, ground, and surface water is a potential threat to human health and the natural environment. Exploring microbial diversity in contaminated habitats has been a compelling and challenging endeavor. This is primarily because a significant portion of the microbial community is either non-cultivable or their growth requirements are not well-understood. Metagenomic DNA was obtained from two distinct samples, PPS-1 and PPS-2, which were contaminated with a wide range of toxic and recalcitrant pollutants. In this study, the V3 and V4 hypervariable regions of the 16S rRNA gene were amplified with the pair of universal bacterial primers to profile the bacterial community. The 16S rRNA gene is approximately 1,500 base pairs long and includes nine hypervariable regions of varying conservation (V1-V9) (Tringe and Hugenholtz, 2008; Kim et al., 2011). The hypervariable regions of 16S rRNA gene demonstrate considerable sequence diversity among different bacteria. The use of primer pairs targeting the hypervariable region of the 16S rRNA gene may bias the estimates of bacterial abundance toward certain phyla (Tremblay et al., 2015). This can be mitigated by using primer pairs that target the V3–V4 hypervariable regions of the 16S rRNA gene.

Such primer pairs have been reported to capture the true range of bacterial phyla and thus are effective for Illumina MiSeq analyses of microbial communities that inhabit complex environments (Klindworth et al., 2013; Vargas-Albores et al., 2019; Fadeev et al., 2021). The V3–V4 hypervariable region provides a broad taxonomic range of bacteria, thus capturing more microbial diversity with decreased taxonomic bias (Rintala et al., 2017; Thijs et al., 2017; Vargas-Albores et al., 2019; Oyewusi et al., 2021). Based on a detailed literature survey of studies focusing on microbial community analysis from contaminated environments, we choose only V3-V4 hypervariable regions of the 16S rRNA gene for bacterial community analysis. After generation of first amplicon, the Miseq library was developed. In this study, HTS analysis for metagenome was performed using an Illumina MiSeq platform using a 2× 300 pair-end sequencing run to generate the raw reads on a MiSeq sequencer. Paired-end sequencing enables the sequencing of template fragments in both the reverse and forward directions on the MiSeq platform. In our study, a total of 1,53,675 and 1,62,691 raw reads were generated from V-3 and V-4 segment of the 16S rRNA gene derived from PPS-1 and PPS-2, respectively. After quality filtering, the MiSeq sequencing yielded 1,15,665 and 119,386 high-quality paired reads.

#### 4.5.2 Alpha diversity of the bacterial communities

Microbial diversity serves as a crucial ecological indicator, with the belief that higher diversity leads to greater sustainability of microbial communities. To explore this aspect, a HTS analysis was carried out to examine the microbial diversity within the sediment samples, PPS-1 and PPS-2. In the present study, the sediment samples were obtained from two different locations polluted with PPMW to unravel the impact of bacterial community composition and diversity using HTS of the metagenome. The total number of obtained high-quality OTUs per sample was used to generate the rarefaction curves and calculate the α-diversity indices. OTUs are commonly used to group clustered sequence data, where each OTU represents a distinct microbial population in the community (Nguyen et al., 2016). In this study, rarefaction curves were constructed to evaluate whether the sequencing depth adequately captured the entire bacterial diversity within the sediments. Consequently, a rarefaction curve typically illustrates the relationship between the number of OTUs and the number of sequenced reads. As observed in Figure 3, as the number of sequences from a sample increase, the number of OTUs converges toward the true diversity. Rarefaction curves generated at the OUT level demonstrated that all samples reached a plateau. This suggests that the sequencing depth was adequate for a comprehensive characterization of each sample, encompassing a significant portion of the bacterial diversity present in sediments.

Additionally, the relative abundance and alpha diversity were also assessed using metrics, such as Shannon and Simpson indices. Shannon and Simpson indices indicated significant differences in alpha diversity of between PPS-1 and PPS-2. In our study, the level of diversity in bacterial communities in PPS-1 was lower than bacterial diversity of PPS-2 based on Shannon diversity index (Supplementary Table S3). Further, Simpson index values were slightly higher for PPS-2 than PPS-1. It was evident that contaminants had a substantial negative impact on bacterial biodiversity, as indicated by the lower Shannon and Simpson indices. Shannon’s index considers both the abundance and evenness of the species present. Similarly, Simpson’s Diversity Index is a measure of diversity that considers both the number of species present and their relative abundances. Shannon diversity is the most effective measure among the commonly used to measure the diversity indices. The α-diversity indices indicate that microorganisms have adapted to prolonged exposure to both organic and inorganic pollution by altering the structure, diversity, and evenness of their communities. A similar result was also reported by Salam et al. (2019).

#### 4.5.3 Taxonomic composition of bacterial community

Currently, a large portion of bacterial and archaeal taxa across diverse ecosystems remains uncultured, limiting our ability to fully characterize environmental microbial communities using traditional culture techniques. Fortunately, the increasing power of NGS technologies of metagenomics enables us to delve deeper and gain a clearer understanding of the structure, function, and diversity of microbial communities in both engineered and natural environments (Cabreros et al., 2023). In this study, illumina (MiSeq) sequencing of metagenome revealed substantial differences in the bacterial community structure between PPS-1 and PPS-2 at various taxonomic levels, originating from Site-1 and Site-2 (Figure 4). The relative diversity and abundance of bacterial populations in the sediment samples from Site-1 and Site-2 have been visualized as stacked bars in the graph. As a result of Figure 4, the microbial taxonomic distribution study displayed *Proteobacteria* followed by the *Bacteroidetes, Planctomycetes, Acidobacteria, Firmicutes, Actinobacteria, Chloroflexi* and *Verrucomicrobia* as the most predominant bacterial phyla in PPS-1 and PPS-2. *Proteobacteria*, the largest and most metabolically diverse phylum, includes all gram-negative bacteria, encompassing chemolithotrophic, chemoorganotrophic, and phototrophic species, which play crucial roles in nutrient cycling and are often encountered in contaminated environments. Our analysis suggests that *Proteobacteria* may play a crucial role in the detoxification and assimilation of both organic and inorganic substances, including heavy metal contaminants, in sediment samples. Earlier many researchers have reported the dominant phylum as *Proteobacteria* in several waste-contaminated sites, such as solid waste dumping site (Kumar R. et al., 2021), distillery waste (Kumar and Chandra, 2020), antibiotic containing waste (Marathe et al., 2016), tannery wastewater (Ma et al., 2018), electronic waste polluted site (Salam and Varma, 2019) and their important role in pollutant removal. The heavy metals/metalloids and pH significantly affected the abundance and structure of most microorganisms (Zhao et al., 2020; Hu et al., 2021). From the phylum assignment result, it was found that the total number of phyla in the PPS-1 and PPS-2 were 37 and 35, respectively, suggesting that the bacterial diversity in the PPS-1 was lower than the PPS-2 even at the phylum level.

In our study, *Bacteroidetes* was the second-highest represented phylum observed in both sediment samples. Bacteroidetes, as a group, are gram-negative, non-sporulating rod-shaped bacteria, and they are primarily chemoheterotrophs (Willey et al., 2019). These bacteria are known for their role in the digestion of cellulose, proteins, and other macromolecules. They possess the capacity to utilize various polysaccharides and organic chemicals as their sole carbon and nitrogen sources. *Bacteroidetes* have successfully colonized various ecological niches and are closely associated with detoxification, the breakdown of organic matter, and their active participation in the carbon cycle within the environment (Willey et al., 2019). This unique ability positions them as a specialized bacterial community capable of potentially contributing to *in situ* remediation of toxic pollutants.

In this study, the third most abundant phylum, *Planctomycetes*, was detected in PPS-1 and PPS-2, representing Gram-negative bacteria with diversity in cell biology, ecology, and physiology. This study explores that with the phylum *Proteobacteria*, the most abundant classes were *Alphaproteobacteria* followed by *Betaproteobacteria, Gammaproteobacteria*, and *Deltaproteobacteria*. *Alphaproteobacteria* is the second largest class of *Proteobacteria* contains extensive functional diversity of organisms, preferring to grow in environments that have low nutrient concentration (Kersters et al., 2006). However, numerous studies have indicated that *Alphaproteobacteria* usually thrive in organic-rich environments, such as sites contaminated with organic pollution. *Betaproteobacteria*, the third largest class of *Proteobacteria*, share metabolic similarities with *Alphaproteobacteria* (Willey et al., 2019). However, they predominantly utilize substances released from organic decomposition in anoxic habitats. *Alphaproteobacteria* and *Betaproteobacteria* had a more significant contribution to the wastewater or wastewater pollutants, which is in agreement with earlier findings (Desta et al., 2014; Jena et al., 2015).

In our study, *Gammaproteobacteria*, the largest and most diverse class of *Proteobacteria*, was detected. The species within *Gammaproteobacteria* exhibit a range of metabolic and ecological characteristics, comprising facultatively aerobic, nonsporulating, gram-negative rods that are either nonmotile or motile (Williams et al., 2010; Dyksma et al., 2016). Previous studies revealed that *Gammaproteobacteria* has been the most dominant phylum in contaminated soils and play a vital role in degrading various recalcitrant contaminants in polluted sites (Dell’Anno et al., 2021; Yang et al., 2021).

At the genus level, the most abundant genus was *Thiobacillus* followed by *Comamonadaceae*, and a lot of Unidentified bacterial genera. *Thiobacillus* is a rod-shaped, gram-negative chemolithotrophs, that belongs to the class, *Betaproteobacteria* (Willey et al., 2019). This genus is particularly noteworthy due to its capacity as chemolithotrophs, as they can oxidize reduced sulfur compounds like thiosulfate, hydrogen sulfide (H_2_S), elemental sulfur (S^0^), or thiocyanate (-SCN) to serve as electron donors in energy generation (Kuenen and Tuovinen, 1981). *Thiobacillus* has been reported as the dominant genus in effluent treatment plants that treat tannery and pesticide contaminated wastewater (Kim et al., 2014; Fang et al., 2018; Pandit et al., 2021). It’s worth mentioning that certain microorganisms with low relative abundance (less than 1%) but playing crucial roles in degradation were also detected in both PPS-1 and PPS-2. In our study, the sequences that failed to align with the taxonomic database were classified as “unidentified.” It’s noteworthy that the presence of unidentified organisms has been reported previously using Illumina MiSeq framework for the analysis of 16S rRNA V3 and V4 segment of metagenome grown in PPMW (Sharma et al., 2021d; Profiling) and activate sludge containing lignin and chlorophenol (Sharma et al., 2023). In our study, numerous uncultured bacteria were also detected at various levels of taxa. The findings of unculture organisms are in agreement with Sharma et al. (2023), who reported a huge number of unculture microbial communities grown in activated sludge discharged from PPM.

### 4.6 Linking bacterial community structure to habitat

Microbial communities are crucial for bioremediating refractory organic pollutants at contaminated sites. To develop an effective strategy, it’s vital to understand how microbial communities respond to chlorolignin compound degradation. Bacteria show more versatility than fungi in breaking down these compounds, and the sediment samples provide an ecological niche for bacterial growth. Our study shows differences in microbial community structure and dynamics between two sites. Variations in microbial diversity across these sites may stem from differences in sludge physico-chemical parameters, contaminant loads, or environmental conditions (Prabha et al., 2017; Singh et al., 2022). Environmental factors, especially pH, significantly influence bacterial diversity. Soil pH and organic content affect microbial community structure, as they impact ion and trace metal availability (Garcia-Sánchez et al., 2015; Zhen et al., 2019; Stefanowicz et al., 2020). Microbial community structure variations are also linked to environmental conditions and pollutant loads (Mark Ibekwe et al., 2012; Verduzo Garibay et al., 2022). The dominant phyla include *Proteobacteria, Bacteroidetes, Planctomycetes, Acidobacteria, Firmicutes*, and *Actinobacteria*. Specific contaminants influence the local increase of bacterial taxa, supporting bioremediation. The presence of pollutants strongly shapes microbial community composition, with habitat more influential than geographical distance. Future studies should consider physico-chemical factors like pH, organic concentration, and seasonal changes in community structure. Additionally, exploring specific genes favoring survival in harsh environments is warranted. This community contributes to the bioremediation and detoxification of chemicals, both organic and inorganic, as well as chlorinated compounds discharged from the PPMs in the pulp and paper-making process.

### 4.7 Environmental monitoring tool

Metagenomic analysis is a valuable tool for environmental monitoring, enabling comprehensive identification of microbial communities in specific habitats (Feng et al., 2018; Jurelevicius et al., 2022). In this study, bacterial communities in sediment samples were analyzed using amplicon NGS targeting the V3 and V4 regions of the 16S rRNA gene. For assessing ecosystem health, soil and sediment samples, known for their high diversity and varying sensitivities to environmental disturbances, are often employed. However, traditional taxonomic approaches based on morphological characteristics are labor-intensive, costly, and time-consuming for routine biomonitoring. In contrast, metagenomic approaches offer an environmentally friendly and efficient alternative, requiring minimal materials (Lan et al., 2024; Wang et al., 2024). While it’s important to note that in metagenomic analysis, the number of OTUs may not precisely reflect the number of species due to inherent limitations in high-throughput sequencing and analysis; nevertheless, the two are positively correlated (Nilsson et al., 2019). Notably, the application of metagenomic analysis as a biomonitoring tool for paper mill waste-contaminated sediment as a biomonitoring tool has not been previously reported in India. Taking into account various physico-chemical conditions such as heavy metal concentrations, and pH, the metagenomic approach to biomonitoring bacterial community holds great promise for assessing environmental health.

## 5 Conclusion and recommendations

To the best of our knowledge, this is the first study utilizing a metagenomic approach to unravel the bacterial communities profile growing in sediment contaminated with recalcitrant pollutants discharged from PPMs. The sediment has very high load of chemical contaminants as revealed by GC–MS analysis, unveiling the presence of numerous refractory organic pollutants, such stigmasterol, β-sitosterol, hexadecanoic acid, octadecanoic acid; 2,4-di-tert-butylphenol; heptacosane; dimethyl phthalate; hexachlorobenzene; 1-decanol,2-hexyl; furane 2,5-dimethyl which has been reported as potential EDCs and mutagenic compounds. Sediment samples, PPS-1 exhibited a notable increase in alpha diversity, as evidenced by the Chao1 richness index. The bacterial community compositions of the PPS-2 samples were greatly diverse, as indicated by OTUs. The sediment samples harbored diverse bacterial phyla, such as *Proteobacteria, Bacteroidetes, Planctomycetes, Acidobacteria, Firmicutes*, and *Actinobacteria*. The most abundant bacterial genus in PPS-1 and PPS-2 was *Thiobacillus*, with a relative abundance of 7.60 and 4.50%, respectively. The present study showed that the recalcitrant contaminants discharged from PPMs significantly affected the bacterial community structure at disposal sites. The results presented in this study offer novel insights into comprehending the characteristics of contaminants, alterations in microbial community structure, and potential functions under pollution stress at chlorolignin waste-polluted sites. These findings hold significance for risk assessments and microbial monitoring efforts. Moreover, this knowledge holds potential for guiding the development of suitable bioremediation techniques aimed at restoring ecological balance in sites polluted by PPMW. The study recommends the detection of a large percentage of genera and species as still unclassified providing avenues for the search of novel genes.

## Data availability statement

Illumina paired-end raw reads data generated in this study were deposited in the National Center for Biotechnology Information (NCBI) sequence read archive (SRA) under accession number PRJNA1035633, available at https://www.ncbi.nlm.nih.gov/sra/PRJNA1035633. The metagenomic project can also be accessed through NCBI under the BioSample Accessions SAMN38095719 and SAMN38095720 with BioProject ID PRJNA1035633 (https://www.ncbi.nlm.nih.gov/bioproject/?term=PRJNA1035633). The metagenomic dataset generated and analyzed during the current study can also be available from Mendeley Data repository available at https://data.mendeley.com/datasets/gpmwyfhbp3/2.

## Author contributions

VK: Conceptualization, Formal Analysis, Investigation, Methodology, Funding acquisition, Software, Validation, Visualization, Writing – original draft, Writing – review & editing. FA: Funding acquisition, Writing – review & editing, PV: Project Administration, Supervision, Resources, Writing – review & editing.

## Footnotes

^1^ https://github.com/usadellab/Trimmomatic

^2^ http://qiime.org/

^3^ http://greengenes.secondgenome.com

^4^ http://rdp.cme.msu.edu/

^5^ https://cran.r-project.org/web/packages/gplots/index.html

## References

[ref1] Abia A. L. K. Alisoltani A. Keshri J. Ubomba-Jaswa E. (2018). Metagenomic analysis of the bacterial communities and their functional profiles in water and sediments of the Apies River, South Africa, as a function of land use. Sci. Total Environ. 616–617, 326–334. doi: 10.1016/j.scitotenv.2017.10.322 29126050

[ref2] An X. Zhong B. Chen G. An W. Xia X. Li H.. (2021). Evaluation of bioremediation and detoxification potentiality for papermaking black liquor by a new isolated thermophilic and alkali-tolerant *Serratia* sp. AXJ-M. J. Hazard. Mater. 406:124285. doi: 10.1016/j.jhazmat.2020.124285 33189463

[ref3] Azzam M. O. Hazaimeh S. A. (2021). Olive mill wastewater treatment and valorization by extraction/concentration of hydroxytyrosol and other natural phenols. Proce. Saf. Environ. Prot. 148, 495–523. doi: 10.1016/j.psep.2020.10.030

[ref4] Balabanič D. Filipič M. Krivograd Klemenčič A. Žegura B. (2021). Genotoxic activity of endocrine disrupting compounds commonly present in paper mill effluents. Sci. Total Environ. 794:148489. doi: 10.1016/j.scitotenv.2021.148489 34217092

[ref5] Beule L. Guerra V. Lehtsaar E. Vaupel A. (2022). Digging deeper: microbial communities in subsoil are strongly promoted by trees in temperate agroforestry systems. Plant Soil 480, 423–437. doi: 10.1007/s11104-022-05591-2

[ref6] Bhatti S. Richards R. McGinn P. (2021). Screening of two freshwater green microalgae in pulp and paper mill wastewater effluents in Nova Scotia, Canada. Water Sci. Technol. 83, 1483–1498. doi: 10.2166/wst.2021.001 33767052

[ref7] Böer S. I. Hedtkamp S. I. Van Beusekom J. E. Fuhrman J. A. Boetius A. Ramette A. (2009). Time-and sediment depth-related variations in bacterial diversity and community structure in subtidal sands. ISME J. 3, 780–791. doi: 10.1038/ismej.2009.29 19340087

[ref8] Bolger A. M. Lohse M. Usadel B. (2014). Trimmomatic: a flexible trimmer for Illumina sequence data. Bioinformatics 30, 2114–2120. doi: 10.1093/bioinformatics/btu170 24695404 PMC4103590

[ref9] Bui H. N. Chen Y. C. Pham A. T. Ng S. L. Lin K. Y. A. Viet Nguyen N. Q.. (2022). Life cycle assessment of paper mill wastewater: a case study in Viet Nam. Water Sci. Technol. 85, 1522–1537. doi: 10.2166/wst.2022.049 35290229

[ref10] Cabreros C. Vermi M. Corpuz A. Castrogiovanni F. Borea L. Sandionigi A.. (2023). Unraveling microbial community by next-generation sequencing in living membrane bioreactors for wastewater treatment. Sci. Total Environ. 886:163965. doi: 10.1016/j.scitotenv.2023.163965 37156389

[ref11] Caporaso J. G. Kuczynski J. Stombaugh J. Bittinger K. Bushman F. D. Costello E. K.. (2010). QIIME allows analysis of high throughput community sequencing data. Nat. Methods 7, 335–336. doi: 10.1038/nmeth.f.303 20383131 PMC3156573

[ref12] Castro A. J. G. Baptista I. E. de Moura K. R. S. Padilha F. Tonietto J. de Souza A. Z. P.. (2018). Exposure to a Brazilian pulp mill effluent impacts the testis and liver in the zebrafish. Compar. Biochem. Physiol. Part C Toxicol. Pharmacol. 206–207, 41–47. doi: 10.1016/j.cbpc.2018.02.005 29499384

[ref13] Central Pulp Paper Research Institute (2021). Annual Report. Available at: https://dpiit.gov.in/sites/default/files/CPPRI_annual_report_20-21_11May2023.pdf).

[ref14] Chandra R. Yadav S. Yadav S. (2017). Phytoextraction potential of heavy metals by native wetland plants growing on chlorolignin containing sludge of pulp and paper industry. Ecol. Eng. 98, 134–145. doi: 10.1016/j.ecoleng.2016.10.017

[ref15] Chen J. S. Hussain B. Tsai H. C. Nagarajan V. Koner S. Hsu B. M. (2023). Analysis and interpretation of hot springs water, biofilms, and sediment bacterial community profiling and their metabolic potential in the area of Taiwan geothermal ecosystem. Sci. Total Environ. 856:159115. doi: 10.1016/j.scitotenv.2022.159115 36181827

[ref16] Choudhary A. K. Kumar S. Sharma C. (2012). Removal of chlorophenolics from pulp and paper mill wastewater through constructed wetland. Water Environ. Res. 85, 54–62. doi: 10.2175/106143012x13415215907419 23409454

[ref17] Chow Y. N. Foo K. Y. (2023). Insights into the per-and polyfluoroalkyl substances- contaminated paper mill processing discharge: detection, phytotoxicity, bioaccumulative profiling, and health risk verification. J. Clean. Prod. 384:135478. doi: 10.1016/j.jclepro.2022.135478

[ref18] Christianson-Heiska I. L. Haavisto T. Paranko J. Bergelin E. Isomaa B. (2008). Effects ofthewood extractives dehydroabietic acid and betulinol on reproductive physiology of zebrafish (*Danio rerio*)—a two-generation study. Aquat. Toxicol. 86, 388–396. doi: 10.1016/j.aquatox.2007.12.001 18207254

[ref19] Coimbra E. C. L. Mounteer A. H. do Carmo A. L. V. Michielsen M. J. F. Tótola L. A. Guerino J. P. F.. (2021). Electrocoagulation of Kraft pulp bleaching filtrates to improve biotreatability. Process. Saf. Environ. Prot. 147, 346–355. doi: 10.1016/j.psep.2020.09.039

[ref20] Conte F. Casalini G. Prati L. Ramis G. Rossetti I. (2022). Photoreforming of model carbohydrate mixtures from pulping industry wastewaters. Int. J. Hydrog. Energy 47, 41236–41248. doi: 10.1016/j.ijhydene.2021.12.260

[ref21] Cook D. L. LaFleur L. Parrish A. Jones J. Hoy D. (1997). Characterization of plant sterols from 22 US pulp and paper mills. Water Sci. Technol. 35, 297–303. doi: 10.1016/S0273-1223(96)00944-4

[ref22] Costigan S. L. Werner J. Ouellet J. D. Hill L. G. Law R. D. (2012). Expression profiling and gene ontology analysis in fathead minnow (*Pimephales promelas*) liver following exposure to pulp and paper mill effluents. Aquat. Toxicol. 122–123, 44–55. doi: 10.1016/j.aquatox.2012.05.011 22728206

[ref23] Dagar S. Singh S. K. Gupta M. K. (2022). Economics of advanced technologies for wastewater treatment: evidence from pulp and paper industry. Front. Environ. Sci. 10:960639. doi: 10.3389/fenvs.2022.960639

[ref24] Dell’Anno F. Rastelli E. Tangherlini M. Corinaldesi C. Sansone C. Brunet C.. (2021). Highly contaminated marine sediments can host rare bacterial taxa potentially useful for bioremediation. Front. Microbiol. 12:584850. doi: 10.3389/fmicb.2021.584850 33732217 PMC7956957

[ref25] Demkina A. Slonova D. Mamontov V. Konovalova O. Yurikova D. Rogozhin V.. (2023). Benchmarking DNA isolation methods for marine metagenomics. Sci. Rep. 13:22138 (2023). doi: 10.1038/s41598-023-48804-z 38092853 PMC10719357

[ref26] Deshmukh N. S. Lapsiya K. L. Savant D. V. Chiplonkar S. A. Yeole T. Y. Dhakephalkar P. K.. (2009). Upflow anaerobic filter for the degradation of adsorbable organic halides (AOX) from bleach composite wastewater of pulp and paper industry. Chemosphere 75, 1179–1185. doi: 10.1016/j.chemosphere.2009.02.042 19327815

[ref27] Desta A. F. Assefa F. Leta S. Stomeo F. Wamalwa M. Njahira M.. (2014). Microbial community structure and diversity in an integrated system of anaerobic-aerobic reactors and a constructed wetland for the treatment of tannery wastewater in Modjo, Ethiopia. PLoS One 9:e0115576. doi: 10.1371/journal.pone.0115576 PMC4277355 25541981

[ref28] Dyksma S. Bischof K. Fuchs B. M. Hoffmann K. Meier D. Meyerdierks A.. (2016). Ubiquitous Gammaproteobacteria dominate dark carbon fixation in coastal sediments. ISME J. 10, 1939–1953. doi: 10.1038/ismej.2015.257 26872043 PMC4872838

[ref29] Eskelinen K. Särkkä H. Kurniawan T. A. Sillanpää M. E. T. (2010). Removal of recalcitrant contaminants from bleaching effluents in pulp and paper mills using ultrasonic irradiation and Fenton-like oxidation, electrochemical treatment, and/or chemical precipitation: a comparative study. Desalination 255, 179–187. doi: 10.1016/j.desal.2009.12.024

[ref30] Ettinger M. Ruchhoft C. Lishka R. (1951). Sensitive 4-aminoantipyrine method for phenolic compounds. Analy. Chem. 23, 1783–1788. doi: 10.1021/ac60060a019

[ref31] Fadeev E. Cardozo-Mino M. G. Rapp J. Z. Bienhold C. Salter I. Salman-Carvalho V.. (2021). Comparison of two 16S rRNA primers (V3–V4 and V4–V5) for studies of Arctic microbial communities. Front. Microbiol. 12:637526. doi: 10.3389/fmicb.2021.637526 33664723 PMC7920977

[ref32] Fan L. Sun F. Yang Z. (2022). Metagenomic analyses reveal nitrogen metabolism responses to copper and chromium contamination in sludge-based microbial communities. J. Water Process Eng. 49:102951. doi: 10.1016/j.jwpe.2022.102951

[ref33] Fang H. Zhang H. Han L. Mei J. Ge Q. Long Z.. (2018). Exploring bacterial communities and biodegradation genes in activated sludge from pesticide wastewater treatment plants via metagenomic analysis. Environ. Pollut. 243, 1206–1216. doi: 10.1016/j.envpol.2018.09.080 30267917

[ref34] Feng G. Xie T. Wang X. Bai J. Tang L. Zhao H.. (2018). Metagenomic analysis of microbial community and function involved in cd-contaminated soil. BMC Microbiol. 18:11. doi: 10.1186/s12866-018-1152-5 29439665 PMC5812035

[ref35] Feranchuk S. Belkova N. Potapova U. Kuzmin D. Belikov S. (2018). Evaluating the use of diversity indices to distinguish between microbial communities with different traits. Res. Microbiol. 169, 254–261. doi: 10.1016/j.resmic.2018.03.004 29800679

[ref36] Fernandes L. Lucas M. S. Maldonado M. I. Oller I. Sampaio A. (2014). Treatment of pulp mill wastewater by *Cryptococcus podzolicus* and solar photo-Fenton: a case study. Chem. Eng. J. 245, 158–165. doi: 10.1016/j.cej.2014.02.043

[ref37] FPAC. (2009) FPAC sustainability report. Forest Products Association of Canada: Ottawa, ON, Canada.

[ref38] Fu L. Xie R. Ma D. Zhang M. Liu L. (2023). Variations in soil microbial community structure and extracellular enzymatic activities along a forest–wetland ecotone in high-latitude permafrost regions. Ecol. Evolut. 13:e10205. doi: 10.1002/ece3.10205 PMC10269122 37332520

[ref39] Garcia-Sánchez M. Garcia-Romera I. Cajthaml T. Tlustoš P. Száková J. (2015). Changes in soil microbial community functionality and structure in a metal-polluted site: the effect of digestate and fly ash applications. J. Environ. Manag. 162, 63–73. doi: 10.1016/j.jenvman.2015.07.042 26225934

[ref40] Guo J. Ni B. J. Han X. Chen X. Bond P. Peng Y.. (2017). Unraveling microbial structure and diversity of activated sludge in a full-scale simultaneous nitrogen and phosphorus removal plant using metagenomic sequencing. Enzy. Microbial. Technol. 102, 16–25. doi: 10.1016/j.enzmictec.2017.03.009 28465056

[ref41] Hajdu-Rahkama R. Puhakka J. A. (2022). High tolerance of chemolithoautotrophic Sulphur oxidizing bacteria towards pulp and paper mill wastewaters and their organic constituents supporting Sulphur recovery in alkaline conditions. Chem. Eng. J. 450:137972. doi: 10.1016/j.cej.2022.137972

[ref42] Han D. Gao P. Li R. Tan P. Xie J. Zhang R.. (2020). Multicenter assessment of microbial community profiling using 16S rRNA gene sequencing and shotgun metagenomic sequencing. J. Adv. Res. 26, 111–121. doi: 10.1016/j.jare.2020.07.010 33133687 PMC7584675

[ref43] Haq I. Kalamdhad A. S. Pandey A. (2022). Genotoxicity evaluation of paper industry wastewater prior and post-treatment with laccase producing *Pseudomonas putida* MTCC 7525. J. Clean. Prod. 342:130981. doi: 10.1016/j.jclepro.2022.130981

[ref44] Hoovield M. Heederick D. J. Kogevinas M. Boffetta P. Needham L. L. Patterson D. G. Jr.. (1998). Second follow-up of a Dutch cohort occupationally exposed to phenoxyherbicides, chlorophenols, and contaminants. Amer. J. Epidemiol. 9, 891–901. doi: 10.1093/oxfordjournals.aje.a009543 9583720

[ref45] Hu X. Wang J. Lv Y. Liu X. Zhong J. Cui X.. (2021). Effects of heavy metals/metalloids and soil properties on microbial communities in farmland in the vicinity of a metals smelter. Front. Microbiol. 12:707786. doi: 10.3389/fmicb.2021.707786 34489896 PMC8417379

[ref46] Jena J. Kumar R. Dixit A. Pandey S. Das T. (2015). Evaluation of simultaneous nutrient and COD removal with polyhydroxybutyrate (PHB) accumulation using mixed microbial consortia under anoxic condition and their bioinformatics analysis. PLoS One 10:e0116230. doi: 10.1371/journal.pone.0116230 25689047 PMC4331290

[ref47] Jurelevicius D. Pereira R. D. S. da Mota F. F. Cury J. C. de Oliveira I. C. Rosado A. S.. (2022). Metagenomic analysis of microbial communities across a transect from low to highly hydrocarbon-contaminated soils in King George Island, maritime Antarctica. Geobiology 20, 98–111. 34545693 10.1111/gbi.12472

[ref48] Kalra Y.P. Maynard D.G. (1991). Methods manual for forest soil and plant analysis, information report NOR-X-319 forestry Canada. Northern Forest Centre: Edmonton.

[ref49] Kaur D. Bhardwaj N. K. Lohchab R. K. (2018). A study on pulping of rice straw and impact of incorporation of chlorine dioxide during bleaching on pulp properties and effluents characteristics. J. Clean. Prod. 170, 174–182. doi: 10.1016/j.jclepro.2017.09.111

[ref50] Kersters K. De Vos P. Gillis M. Swings J. Vandamme P. Stackebrandt E. (2006). “Introduction to the Proteobacteria” in The prokaryotes. eds. Dworkin M. Falkow S. Rosenberg E. Schleifer K. H. Stackebrandt E. (New York, NY: Springer)

[ref51] Kim I. S. Ekpeghere K. I. Ha S. Y. Kim B. S. Song B. Kim J. T.. (2014). Full-scale biological treatment of tannery wastewater using the novel microbial consortium BM-S-1. J. Environ. Sci. Health A Tox. Hazard. Subst. Environ. Eng. 49, 355–364. doi: 10.1080/10934529.2014.846707 24279627

[ref52] Kim M. Morrison M. Yu Z. (2011). Evaluation of different partial 16S rRNA gene sequence regions for phylogenetic analysis of microbiomes. J. Microbiol. Method. 84, 81–87. doi: 10.1016/j.mimet.2010.10.020 21047533

[ref53] Klindworth A. Pruesse E. Schweer T. Peplies J. Quast C. Horn M.. (2013). Evaluation of general 16S ribosomal RNA gene PCR primers for classical and next-generation sequencing-based diversity studies. Nucl. Acid. Res. 41:e1. doi: 10.1093/nar/gks808 PMC3592464 22933715

[ref54] Kuenen J. G. Tuovinen O. H. (1981). “The genera Thiobacillus and Thiomicrospira” in The prokaryotes: a handbook on habitats, isolation, and identification of bacteria. eds. Starr M. P. Stolp H. Trüoper H. G. Balow A. Schlegel H. G. (Berlin, Heidelberg: Springer Berlin Heidelberg), 1023–1036.

[ref55] Kumar B. (2014). Quick and easy method for determination of priority phenolic compounds in water and wastewater. J. Xenobiot. 4:4680. doi: 10.4081/xeno.2014.4680

[ref56] Kumar V. Ameen F. Islam M. A. Agrawal S. Motghare A. Dey A.. (2022). Evaluation of cytotoxicity and genotoxicity effects of refractory pollutants of untreated and biomethanated distillery effluent using *Allium cepa*. Environ. Pollut. 300:118975. doi: 10.1016/j.envpol.2022.118975 35157935

[ref57] Kumar V. Chandra R. (2020). Metagenomics analysis of rhizospheric bacterial communities of *Saccharum arundinaceum* growing on organometallic sludge of sugarcane molasses-based distillery. 3 Biotech 10:316. doi: 10.1007/s13205-020-02310-5 PMC7314892 32612900

[ref58] Kumar A. Chandra R. (2021). Biodegradation and toxicity reduction of pulp paper mill wastewater by isolated laccase producing *Bacillus cereus* AKRC03. Clean. Eng. Technol. 4:100193. doi: 10.1016/j.clet.2021.100193

[ref59] Kumar V. Chopra A. K. (2012). Effects of paper mill effluent irrigation on gronomical characteristics of *Vigna radiata* (L.) in two different seasons. Communic. Soil Sci. Plant Analy. 43, 16, 2142–2166. doi: 10.1080/00103624.2012.697236

[ref60] Kumar R. Pandit P. Kumar D. Patel Z. Pandya L. Kumar M.. (2021). Landfill microbiome harbour plastic degrading genes: a metagenomic study of solid waste dumping site of Gujarat, India. Sci. Total Environ. 779:146184. doi: 10.1016/j.scitotenv.2021.146184 33752005

[ref61] Kumar V. Shahi S. K. Ferreira L. F. R. Bilal M. Biswas J. K. Bulgariu L. (2021). Detection and characterization of refractory organic and inorganic pollutants discharged in biomethanated distillery effluent and their phytotoxicity, cytotoxicity, and genotoxicity assessment using *Phaseolus aureus* L. and *Allium cepa* L. Environ. Res. 201:111551. doi: 10.1016/j.envres.2021.111551 34192556

[ref62] Kumar A. Singh A. K. Chandra R. Singh A. K. Chandra R. (2020). Comparative analysis of residual organic pollutants from bleached and unbleached paper mill wastewater and their toxicity on *Phaseolus aureus* and *Tubifex tubifex*. Urban Water J. 17, 860–870. doi: 10.1080/1573062X.2020.1836238

[ref63] Kumar V. Singh J. Kumar P. (2019). Regression models for removal of heavy metals by water hyacinth (*Eichhornia crassipes*) from wastewater of pulp and paper processing industry. Environ. Sustain. 3, 35–44. doi: 10.1007/s42398-019-00093-x

[ref64] Kumar R. Singh A. Maurya A. Yadav P. Yadav A. Chowdhary P.. (2022). Effective bioremediation of pulp and paper mill wastewater using *Bacillus cereus* as a possible Kraft lignin-degrading bacterium. Bioresour. Technol. 352:127076. doi: 10.1016/j.biortech.2022.127076 35351569

[ref65] Kumar A. Srivastava N. K. Gera P. (2021). Removal of color from pulp and paper mill wastewater- methods and techniques- a review. J. Environ. Manag. 298:113527. doi: 10.1016/j.jenvman.2021.113527 34411799

[ref66] Kumar V. Verma P. (2023). A critical review on environmental risk and toxic hazards of refractory pollutants discharged in chlorolignin waste of pulp and paper mills and their remediation approaches for environmental safety. Environ. Res. 236:116728. doi: 10.1016/j.envres.2023.116728 37495063

[ref67] Lan W. Liu H. Weng R. Zeng Y. Lou J. Xu H.. (2024). Microbial community of municipal drinking water in Hangzhou using metagenomic sequencing. Environ. Pollut. 342:123066. doi: 10.1016/j.envpol.2023.123066 38048871

[ref68] Lee Y. Y. Lee S. Y. Cho K. S. (2023). Phytoremediation and bacterial community dynamics of diesel- and heavy metal-contaminated soil: Long-term monitoring on a pilot scale. Int. Biodeterior. Biodegrad. 183:105642. doi: 10.1016/j.ibiod.2023.105642

[ref69] Liang Y. Jiao C. Pan L. Zhao T. Liang J. Xiong J.. (2021). Degradation of chlorine dioxide bleaching wastewater and response of bacterial community in the intimately coupled system of visible-light photocatalysis and biodegradation. Environ. Res. 195:110840. doi: 10.1016/j.envres.2021.110840 33587946

[ref70] Ma X. Wu C. Huang J. Zhou R. Shi B. (2018). Microbial community of tannery wastewater involved in nitrification revealed by Illumina MiSeq sequencing. J. Microbiol. Biotechnol. 28, 1168–1177. doi: 10.4014/jmb.1712.12054 29975994

[ref1002] MacLatchy D. L. Van Der Kraak G. J. (1995). The Phytoestrogen β-Sitosterol alters the reproductive endocrine status of Goldfish. Toxicol. Appl. Pharmacol. 134, 305–312. doi: 10.1006/taap.1995.1196 7570607

[ref71] Maniatis T. Fritsch E. F. Sambrook J. (1982) Molecular cloning: a laboratory manual. Cold Spring Harbor: Cold Spring Harbor Laboratory

[ref72] Mapelli F. Vergani L. Terzaghi E. Zecchin S. Raspa G. Marasco R.. (2022). Pollution and edaphic factors shape bacterial community structure and functionality in historically contaminated soils. Microbiol. Res. 263:127144. doi: 10.1016/j.micres.2022.127144 35908425

[ref73] Marathe N. P. Shetty S. A. Shouche Y. S. Larsson D. J. (2016). Limited bacterial diversity within a treatment plant receiving antibiotic-containing waste from bulk drug production. PLoS One 11:e0165914. doi: 10.1371/journal.pone.0165914 27812209 PMC5094703

[ref74] Mark Ibekwe A. Leddy M. B. Bold R. M. Graves A. K. (2012). Bacterial community composition in low-flowing river water with different sources of pollutants. FEMS Microbiol. Ecol. 79, 155–166. doi: 10.1111/j.1574-6941.2011.01205.x 22066546

[ref75] Maurin N. Sayen S. Guillon E. (2023). Gas chromatography–mass spectrometry analysis of organic pollutants in French soils irrigated with agro-industrial wastewater. Front. Environ. Sci. 11:417. doi: 10.3389/fenvs.2023.1125487

[ref76] McElroy A. E. Barron M. G. Beckvar N. Driscoll S. B. K. Meador J. P. Parkerton T. F.. (2011). A review of the tissue residue approach for organic and organometallic compounds in aquatic organisms. Integ. Environ. Assesst. Manag. 7, 50–74. doi: 10.1002/ieam.132 21184569

[ref1001] McMurdie P. J. Holmes S. (2013). Phyloseq: an R package for reproducible interactive analy- sis and graphics of microbiome census data. PLOS ONE 8:e61217. doi: 10.1371/journal.pone.0061217 23630581 PMC3632530

[ref77] Medeiros P. M. (2018). “Gas chromatography–mass spectrometry (GC–MS)” in Encyclopedia of geochemistry. Encyclopedia of earth sciences series. ed. White W. M. (Cham: Springer)

[ref78] Medhi U. J. Talukdar A. K. Deka S. (2011). Impact of paper mill effluent on growth and development of certain agricultural crops. J. Environ. Biol. 32, 185–188. 21882653

[ref79] Mellanen P. Petanen T. Lehtimaki J. Makela S. Bylund G. Holmbom B.. (1996). Wood-derived estrogens: studies in vitro with breast cancer cell lines and in vivo in trout. Toxicol. Appl. Pharmacol. 136, 381–388. doi: 10.1006/taap.1996.0046 8619247

[ref80] Mendes L. W. Tsai S. M. (2014). Variations of bacterial community structure and composition in mangrove sediment at different depths in southeastern Brazil. Diversity 6, 827–843. doi: 10.3390/d6040827

[ref81] Mishra S. Kumar R. Kumar M. (2023). Use of treated sewage or wastewater as an irrigation water for agricultural purposes-environmental, health, and economic impacts. Total Environ. Res. Themes 6:100051. doi: 10.1016/j.totert.2023.100051

[ref82] Mitra S. Chakraborty A. J. Tareq A. M. Emran T. B. Nainu F. Khusro A.. (2022). Impact of heavy metals on the environment and human health: novel therapeutic insights to counter the toxicity. J. King Saud Univer Sci. 34:101865. doi: 10.1016/j.jksus.2022.101865

[ref83] Mittar D. Khanna P. K. Marwaha S. S. Kennedy J. F. (1992). Biobleaching of pulp and paper mill effluents by *Phanerochaete chrysosporium*. J. Chem. Technol. Biotechnol. 53, 81–92. doi: 10.1002/jctb.280530112

[ref84] Möder M. Schrader S. Franck U. Popp P. (1997). Determination of phenolic compounds in waste water by solid-phase micro extraction. Fresenius’ J. Analy. Chem. 357, 326–332. doi: 10.1007/s002160050162

[ref85] Moter A. Göbel U. B. (2000). Fluorescence in situ hybridization (FISH) for direct visualization of microorganisms. J. Microbiol. Methods 41, 85–112. doi: 10.1016/S0167-7012(00)00152-4 10991623

[ref86] Muthukumar B. Parthipan P. AlSalhi M. S. Prabhu N. S. Rao T. N. Devanesan S.. (2022). Characterization of bacterial community in oil-contaminated soil and its biodegradation efficiency of high molecular weight (>C40) hydrocarbon. Chemosphere 289:133168. doi: 10.1016/j.chemosphere.2021.133168 34890617

[ref87] Muyzer G. Smalla K. (1998). Application of denaturing gradient gel electrophoresis (DGGE) and temperature gradient gel electrophoresis (TGGE) in microbial ecology. Antonie Van Leeuwenhoek 73, 127–141. doi: 10.1023/A:1000669317571 9602286

[ref88] Namdarimonfared M. Zilouei H. Tondro H. (2023). Biological hydrogen production from paper mill effluent via dark fermentation in a packed bed biofilm reactor. Fuel 338:127231. doi: 10.1016/j.fuel.2022.127231

[ref89] Nguyen N. P. Warnow T. Pop M. White B. (2016). A perspective on 16S rRNA operational taxonomic unit clustering using sequence similarity. NPJ Biofil. Microbiom. 2:16004. doi: 10.1038/npjbiofilms.2016.4 PMC5515256 28721243

[ref90] Nilsson R. H. Anslan S. Bahram M. Wurzbacher C. Baldrian P. Tedersoo L. (2019). Mycobiome diversity: high-throughput sequencing and identification of fungi. Nat. Rev. Microbiol. 17, 95–109. doi: 10.1038/s41579-018-0116-y 30442909

[ref91] Ning J. Liebich J. Kastner M. Zhou J. Schaffer A. Burauel P. (2009). Different influences of DNA purity indices and quantity on PCR based DGGE and functional gene microarray in soil microbial community study. Appl. Microbiol. Biotechnol. 82, 983–993. doi: 10.1007/s00253-009-1912-0 19247649

[ref92] Oke N. Singh S. Garg A. (2017). A comparative treatment of bleaching wastewater by physicochemical processes. Water Sci. Technol. 76, 2367–2379. doi: 10.2166/wst.2017.355 29144295

[ref93] Olson N. D. Morrow J. B. (2012). DNA extract characterization process for microbial detection methods development and validation. BMC. Res. Notes 5, 1–14. doi: 10.1186/1756-0500-5-668 23206278 PMC3599793

[ref94] Orrego R. Guchardi J. Hernandez V. Krause R. Roti L. Armour J.. (2009). Pulp and paper mill effluent treatments have differential endocrine-disrupting effects on rainbow trout. Environ. Toxicol. Chem. 28, 181–188. doi: 10.1897/08-191.1 18717619

[ref95] Orrego R. Guchardi J. Krause R. Holdway D. (2010). Estrogenic and anti-estrogenic effects of wood extractives present in pulp and paper mill effluents on rainbow trout. Aquat. Toxicol. 99, 160–167. doi: 10.1016/j.aquatox.2010.04.016 20483492

[ref96] Orrego R. Hewitt L. M. McMaster M. Chiang G. Quiroz M. Munkittrick K.. (2019). Assessing wild fish exposure to ligands for sex steroid receptors from pulp and paper mill effluents in the Biobio River Basin, Central Chile. Ecotoxicol. Environ. Saf. 171, 256–263. doi: 10.1016/j.ecoenv.2018.12.092 30612013

[ref97] Osborn A. M. Moore E. R. Timmis K. N. (2000). An evaluation of terminal-restriction fragment length polymorphism (T-RFLP) analysis for the study of microbial community structure and dynamics. Environ. Microbiol. 2, 39–50. doi: 10.1046/j.1462-2920.2000.00081.x 11243261

[ref98] Oyewusi H. A. Abdul Wahab R. Edbeib M. F. Mohamad M. A. N. Abdul Hamid A. A. Kaya Y.. (2021). Functional profiling of bacterial communities in Lake Tuz using 16S rRNA gene sequences. Biotechnol. Biotechnol. Equip. 35, 1–10. doi: 10.1080/13102818.2020.1840437

[ref99] Pandit P. R. Kumar R. Kumar D. Patel Z. Pandya L. Kumar M.. (2021). Deciphering the black box of microbial community of common effluent treatment plant through integrated metagenomics: tackling industrial effluent. J. Environ. Manag. 289:112448. doi: 10.1016/j.jenvman.2021.112448 33831764

[ref100] Pearl I. A. Benson H. K. (1990). The determination of lignin in sulphide pulping liquor. Paper Trade J. 111, 35–36.

[ref101] Prabha S. Gogoi A. Mazumder P. Ramanathan A. L. Kumar M. (2017). Assessment of the impact of textile effluents on microbial diversity in Tirupur district, Tamil Nadu. Appl. Water Sci. 7, 2267–2277. doi: 10.1007/s13201-016-0394-3

[ref102] Prada P. Brunel B. Reffuveille F. Gangloff S. C. (2022). Technique evolutions for microorganism detection in complex samples: a review. Appl. Sci. 12:5892. doi: 10.3390/app12125892

[ref103] Prakash A. A. Rajasekar A. Sarankumar R. K. AlSalhi M. S. Devanesan S. Aljaafreh M. J.. (2021). Metagenomic analysis of microbial community and its role in bioelectrokinetic remediation of tannery contaminated soil. J. Hazard. Mater. 412:125133. doi: 10.1016/j.jhazmat.2021.125133 33524735

[ref104] Qian F. Huang X. Su X. Bao Y. (2022). Responses of microbial communities and metabolic profiles to the rhizosphere of *Tamarix ramosissima* in soils contaminated by multiple heavy metals. J. Hazard. Mater. 438:129469. doi: 10.1016/j.jhazmat.2022.129469 35820335

[ref105] Rajput H. Changotra R. Kumar V. Dhir A. (2020). Photoelectrocatalytic treatment of recalcitrant compounds and bleach stage pulp and paper mill effluent using Au-TiO_2_ nanotube electrode. Chem. Eng. J. 408:127287. doi: 10.1016/j.cej.2020.127287

[ref106] Ranjard L. Poly F. Nazaret S. (2000). Monitoring complex bacterial communities using culture-independent molecular techniques: application to soil environment. Res. Microbiol. 151, 167–177. doi: 10.1016/S0923-2508(00)00136-4 10865943

[ref107] Ratia H. Vuori K. M. Oikari A. (2012). Caddis larvae (Trichoptera, Hydropsychidae) indicate delaying recovery of a watercourse polluted by pulp and paper industry. Ecol. Indic. 15, 217–226. doi: 10.1016/j.ecolind.2011.09.015

[ref108] Rintala A. Pietilä S. Munukka E. Eerola E. Pursiheimo J.-P. Laiho A.. (2017). Gut microbiota analysis results are highly dependent on the 16S rRNA gene target region, whereas the impact of DNA extraction is minor. J. Biomol. Tech. 28, 19–30. doi: 10.7171/jbt.17-2801-003 28260999 PMC5330390

[ref109] Salam L. N. Shomope H. Ummi Z. Bukar F. (2019). Mercury contamination imposes structural shift on the microbial community of an agricultural soil. Bullet. Nat. Res. Cent 43:163. doi: 10.1186/s42269-019-0208-5

[ref110] Salam M. Varma A. (2019). Bacterial community structure in soils contaminated with electronic waste pollutants from Delhi NCR, India. Elect. J. Biotechnol. 41, 72–80. doi: 10.1016/j.ejbt.2019.07.003

[ref111] Selvi A. AlSalhi M. S. Devanesan S. Maruthamuthu M. K. Mani P. Rajasekar A. (2021). Characterization of biospheric bacterial community on reduction and removal of chromium from tannery contaminated soil using an integrated approach of bio-enhanced electrokinetic remediation. J. Environ. Chem. Eng. 9:106602. doi: 10.1016/j.jece.2021.106602

[ref112] Sharma P. Chandra R. Yadav S. (2023). Quantification of microbial communities in activated sludge containing lignin and chlorophenol from the pulp and paper industry as determined by 16S rRNA analysis. Bioresour. Technol. Rep. 21:101371. doi: 10.1016/j.biteb.2023.101371

[ref113] Sharma P. Purchase D. Chandra R. (2021a). Residual pollutants in treated pulp paper mill wastewater and their phytotoxicity and cytotoxicity in *Allium cepa*. Environ. Geochem. Health 43, 2143–2164. doi: 10.1007/s10653-020-00730-z 33400008

[ref114] Sharma A. Thakur V. V. Shrivastava A. Jain R. K. Mathur R. M. Gupta R.. (2014). Xylanase and laccase based enzymatic Kraft pulp bleaching reduces adsorbable organic halogen (AOX) in bleach effluents: a pilot scale study. Bioresour. Technol. 169, 96–102. doi: 10.1016/j.biortech.2014.06.066 25036336

[ref115] Sharma P. Tripathi S. Chandra R. (2021b). Metagenomic analysis for profiling of microbial communities and tolerance in metal-polluted pulp and paper industry wastewater. Bioresour. Technol. 324:124681. doi: 10.1016/j.biortech.2021.124681 33454444

[ref116] Sharma P. Tripathi S. Chandra R. (2021c). Highly efficient phytoremediation potential of metal and metalloids from the pulp paper industry waste employing *Eclipta alba* (L) and *Alternanthera philoxeroide* (L): biosorption and pollution reduction. Bioresour. Technol. 319:124147. doi: 10.1016/j.biortech.2020.124147 32992272

[ref117] Sharma P. Tripathi S. Vadakedath N. Chandra R. (2021d). In-situ toxicity assessment of pulp and paper industry wastewater on *Trigonella foenum-graecum* L: potential source of cytotoxicity and chromosomal damage. Environ. Technol. Innov. 21:101251. doi: 10.1016/j.eti.2020.101251

[ref118] Singh P. K. Deshbhratar P. B. Ramteke D. S. (2012). Monitoring colour and COD removal capacity of soil and assessment of growth performance of crop growth with paper mill wastewater—a lysimeter study. Chem. Eng. J. 187, 1–9. doi: 10.1016/j.cej.2010.09.042

[ref119] Singh A. K. Kumar A. Chandra R. (2020). Detection of refractory organic pollutants from pulp paper mill effluent and their toxicity on *Triticum aestivum*; brassica campestris and tubifex-tubifex. J. Experimen. Biol. Agric. Sci. 8, 663–675. doi: 10.18006/2020.8(5).663.675

[ref120] Singh J. Kumar V. Kumar P. Kumar P. Yadav K. K. Cabral-Pinto M. M. S.. (2021). An experimental investigation on phytoremediation performance of water lettuce (*Pistia stratiotes* L.) for pollutants removal from paper mill effluent. Water Environ. Res. 93, 1543–1553. doi: 10.1002/wer.1536 33565675

[ref121] Singh A. Varma A. Prasad R. Porwal S. (2022). Bioprospecting uncultivable microbial diversity in tannery effluent contaminated soil using shotgun sequencing and bio-reduction of chromium by indigenous chromate reductase genes. Environ. Res. 215:114338. doi: 10.1016/j.envres.2022.114338 36116499

[ref122] Sonkar M. Kumar M. Dutt D. Kumar V. (2019). Treatment of pulp and paper mill effluent by a novel bacterium *Bacillus* sp. IITRDVM-5 through a sequential batch process. Biocatal. Agric. Biotechnol. 20:101232. doi: 10.1016/j.bcab.2019.101232

[ref123] Špánik I. Machyňáková A. (2018). Recent applications of gas chromatography with high-resolution mass spectrometry. J. Sep. Sci. 41, 163–179. doi: 10.1002/jssc.201701016 29111584

[ref124] Stefanowicz A. M. Kapusta P. Zubek S. Stanek M. Woch M. W. (2020). Soil organic matter prevails over heavy metal pollution and vegetation as a factor shaping soil microbial communities at historical Zn–Pb mining sites. Chemosphere 240:124922. doi: 10.1016/j.chemosphere.2019.124922 31563718

[ref125] Thijs S. Op De Beeck M. Beckers B. Truyens S. Stevens V. Van Hamme J. D.. (2017). Comparative evaluation of four bacteria-specific primer pairs for 16S rRNA gene surveys. Front. Microbiol. 8:494. doi: 10.3389/fmicb.2017.00494 28400755 PMC5368227

[ref126] Tong T. Li R. Chai M. Wang Q. Yang Y. Xie S. (2021). Metagenomic analysis of microbial communities continuously exposed to bisphenol a in mangrove rhizosphere and non-rhizosphere soils. Sci. Total Environ. 792:148486. doi: 10.1016/j.scitotenv.2021.148486 34465064

[ref127] Tremblay J. Singh K. Fern A. Kirton E. S. He S. Woyke T.. (2015). Primer and platform effects on 16S rRNA tag sequencing. Front. Microbiol. 6:771. doi: 10.3389/fmicb.2015.00771 26300854 PMC4523815

[ref128] Tremblay L. A. Van Der Kraak G. J. (1998). Use of a series of homologous in vitro and in vivo assays to evaluate the endocrine modulating actions of beta-sitosterol in rainbow trout. Aquat. Toxicol. 43, 149–162. doi: 10.1016/S0166-445X(98)00051-4

[ref129] Tringe S. G. Hugenholtz P. (2008). A renaissance for the pioneering 16S rRNA gene. Curr. Opin. Microbiol. 11, 442–446. doi: 10.1016/j.mib.2008.09.011 18817891

[ref130] Tripathi S. Yadav S. Purchase D. Singh K. al-Shwaiman H. A. Chandra R. (2022). Characterization of persistent organic pollutants and culturable and non-culturable bacterial communities in pulp and paper sludge after secondary treatment. Chemosphere 295:133892. doi: 10.1016/j.chemosphere.2022.133892, PMID: 35134397

[ref131] Tripathy A. P. Dixit P. K. Panigrahi A. K. (2022). Impact of effluent of Pulp & Paper industry on the flora of river basin at Jaykaypur, Odisha, India and its ecological implications. Environ. Res. 204:111769. doi: 10.1016/j.envres.2021.111769 34419471

[ref132] USEPA (1996). Acid digestion of sediments, sludges, and soils. Available at: https://www.epa.gov/sites/production/files/2015-06/documents/epa-3050b.pdf.

[ref133] USEPA (2012). U.S. Environmental Protection Agency Endocrine Disruptor Screening Program Universe of Chemicals. United States Environmental Protection Agency: Washington, DC.

[ref134] Vandeputte D. Tito R. Y. Vanleeuwen R. Falony G. Raes J. (2017). Practical considerations for large-scale gut microbiome studies. FEMS Microbiol. Rev. 41, S154–S167. doi: 10.1093/femsre/fux027 28830090 PMC7207147

[ref135] Vargas-Albores F. Martınez-Cordova L. R. Martınez-Porchas M. Calderón K. Lago-Lestón A. (2019). (2019). Functional metagenomics: a tool to gain knowledge for agronomic and veterinary sciences. Biotechnol. Genet. Rev 35, 69–91. doi: 10.1080/02648725.2018.1513230 30221593

[ref136] Verduzo Garibay M. Fernández del Castillo A. de Anda J. Senés-Guerrero C. Gradilla-Hernández M. S. (2022). Structure and activity of microbial communities in response to environmental, operational, and design factors in constructed wetlands. Int. J. Environ. Sci. Technol. 19, 11587–11612. doi: 10.1007/s13762-021-03719-y

[ref137] Walters K. E. Martiny J. B. H. (2020). Alpha-, beta-, and gamma-diversity of bacteria varies across habitats. PLoS One 15:e0233872. doi: 10.1371/journal.pone.0233872 32966309 PMC7510982

[ref138] Wang H. Chen N. Feng C. (2023). Priming effect and mechanism of nitrate and vanadate removal from agro-industrial waste-based colonizing microbial communities. J. Clean. Prod. 395:136384. doi: 10.1016/j.jclepro.2023.136384

[ref139] Wang H. Gong H. Dai X. Yang M. (2024). Metagenomics reveals the microbial community and functional metabolism variation in the partial nitritation-anammox process: from collapse to recovery. J. Environ. Sci. 135, 210–221. doi: 10.1016/j.jes.2023.01.002 37778796

[ref140] Willey J. Sandman K. Wood D. (2019). Prescott's microbiology. New York: McGraw-Hill. 11th

[ref141] Williams K. P. Gillespie J. J. Sobral B. W. Nordberg E. K. Snyder E. E. Shallom J. M.. (2010). Phylogeny of gammaproteobacteria. J. Bacteriol. 192, 2305–2314. doi: 10.1128/jb.01480-09 20207755 PMC2863478

[ref142] Yadav S. Chandra R. (2018). Detection and assessment of the phytotoxicity of residual organic pollutants in sediment contaminated with pulp and paper mill effluent. Environ. Monit. Assess. 190, 1–15. doi: 10.1007/s10661-018-6947-1 30206720

[ref143] Yang L. Han D. Jin D. Zhang J. Shan Y. Wan M.. (2023). Soil physiochemical properties and bacterial community changes under long-term polycyclic aromatic hydrocarbon stress in situ steel plant soils. Chemosphere 334:138926. doi: 10.1016/j.chemosphere.2023.138926 37182712

[ref144] Yang X. Huan Z. L. Zhao S. P. Xi H. L. (2023). Study on environmental pollution behavior/fate of ammunition soil and microbial remediation of TNT and its intermediates. J. Clean. Prod. 432:139715. doi: 10.1016/j.jclepro.2023.139715

[ref145] Yang X. Lai J. Zhang Y. Luo X. G. Han M. W. Zhao S. P. (2021). Microbial community structure and metabolome profiling characteristics of soil contaminated by TNT, RDX, and HMX. Environ. Pollut. 285:117478. doi: 10.1016/j.envpol.2021.117478, PMID: 34087636

[ref146] Yergeau E. Sanschagrin S. Beaumier D. Greer C. W. (2012). Metagenomic analysis of the bioremediation of diesel-contaminated Canadian high arctic soils. PLoS One 7:e30058. doi: 10.1371/journal.pone.0030058 22253877 PMC3256217

[ref147] Yue W. Genji Y. Bowen W. Yaozu M. Yang Z. Tian M.. (2023). Papermaking wastewater treatment coupled to 2,3-butanediol production by engineered psychrotrophic *Raoultella terrigena*. J. Hazard. Mater. 458:131994. doi: 10.1016/j.jhazmat.2023.131994 37418966

[ref148] Yuliani G. Cha A. L. Garnier G. (2019). UV-induced colour generation of pulp and paper mill effluents as a proxy of ligno-cellulosic biorefinery wastewater. J. Water Process Eng. 29:100781. doi: 10.1016/j.jwpe.2019.100781

[ref149] Zhang X. Li J. Gong J. Kuang Y. He S. Xu J.. (2020). Cleaner approach for medium consistency eucalyptus slab pulp production using ozone bleaching under turbulent mixing. J. Clean. Prod. 276:124201. doi: 10.1016/j.jclepro.2020.124201

[ref150] Zhang C. Nie S. Liang J. Zeng G. Wu H. Hua S.. (2016). Effects of heavy metals and soil physicochemical properties on wetland soil microbial biomass and bacterial community structure. Sci. Tot. Environ. 557, 785–790. doi: 10.1016/j.scitotenv.2016.01.170 27046142

[ref151] Zhao X. Huang J. Zhu X. Chai J. Ji X. (2020). Ecological effects of heavy metal pollution on soil microbial community structure and diversity on both sides of a river around a mining area. Int. J. Environ. Res. Pub. Heal. 17:5680. doi: 10.3390/ijerph17165680 PMC7460318 32781566

[ref152] Zhao H. Zheng W. Zhang S. Gao W. Fan Y. (2021). Soil microbial community variation with time and soil depth in Eurasian steppe (Inner Mongolia, China). Annal. Microbiol. 71:21. doi: 10.1186/s13213-021-01633-9

[ref153] Zhen Z. Wang S. Luo S. Ren L. Liang Y. Yang R.. (2019). Significant impacts of both total amount and availability of heavy metals on the functions and assembly of soil microbial communities in different land use patterns. Front. Microbiol. 10:2293. doi: 10.3389/fmicb.2019.02293 31636621 PMC6788306

[ref154] Zhu D. Wang Z. Zhang Z. (2023). Effects of heavy metal pollution and soil physicochemical properties on the Sphagnum farmland soil microbial community structure in Southern Guizhou, China. Int. J. Phytoremediation 25, 1762–1773. doi: 10.1080/15226514.2023.2191139 36949727

