# Supplementary material for: Unraveling the shift in bacterial communities profile grown in sediments co-contaminated with chlorolignin waste of pulp-paper mill by metagenomics approach
Source: Front Microbiol. 2024 Mar 11;15:1350164. doi: 10.3389/fmicb.2024.1350164 (PMC10961449; doi:10.3389/fmicb.2024.1350164)
Supplement: Supplementary file 1 [file Table_1.docx]

**Table S1** Heavy metals profile of sediment samples collected from two different sites polluted with chlorolignin contaminants of pulp-paper mills

| **Metals** | **Unit** | **Sample** | **Min** | **Max** | **Mean** | **SD** |
| --- | --- | --- | --- | --- | --- | --- |
| Copper | mg kg^−1^ | PPS-1 | 0.009 | 0.009 | 0.009 | 0 |
|  | mg kg^−1^ | PPS-2 | 0.010 | 0.011 | 0.01 | 0.001^ns^ |
| Nickel | mg kg^−1^ | PPS-1 | 0.004 | 0.006 | 0.005 | 0.001 |
|  | mg kg^−1^ | PPS-2 | 0.002 | 0.003 | 0.002 | 0.000^ns^ |
| Manganese | mg kg^−1^ | PPS-1 | 0.078 | 0.079 | 0.078 | 0.000 |
|  | mg kg^−1^ | PPS-2 | 0.051 | 0.062 | 0.056 | 0.005^***^ |
| Chromium | mg kg^−1^ | PPS-1 | 0.014 | 0.016 | 0.015 | 0.001 |
|  | mg kg^−1^ | PPS-2 | 0.009 | 0.011 | 0.009 | 0.001^ns^ |
| Lead | mg kg^−1^ | PPS-1 | 0.007 | 0.009 | 0.008 | 0.001 |
|  | mg kg^−1^ | PPS-2 | 0.005 | 0.008 | 0.006 | 0.001^ns^ |
| Zinc | mg kg^−1^ | PPS-1 | 0.224 | 0.227 | 0.225 | 0.001 |
|  | mg kg^−1^ | PPS-2 | 0.088 | 0.090 | 0.086 | 0.005^***^ |
| Iron | mg kg^−1^ | PPS-1 | 2.121 | 2.126 | 2.124 | 0.002 |
|  | mg kg^−1^ | PPS-2 | 0.715 | 0.802 | 0.764 | 0.044^***^ |
| Arsenic | mg kg^−1^ | PPS-1 | 0.005 | 0.007 | 0.006 | 0.001 |
|  | mg kg^−1^ | PPS-2 | 0.005 | 0.007 | 0.006 | 0.001^ns^ |
| Cadmium | mg kg^−1^ | PPS-1 | 0.000 | 0.009 | 0.003 | 0.005 |
|  | mg kg^−1^ | PPS-2 | BDL | BDL | BDL | -- |
| Cobalt | mg kg^−1^ | PPS-1 | 0.001 | 0.003 | 0.001 | 0.001 |
|  | mg kg^−1^ | PPS-2 | 0.000 | 0.000 | 0.000 | 0.000^ns^ |
| Aluminium | mg kg^−1^ | PPS-1 | 3.472 | 3.484 | 3.477 | 0.006 |
|  | mg kg^−1^ | PPS-2 | 32.248 | 2.332 | 22.277 | 17.272^*^ |
| Antimony | mg kg^−1^ | PPS-1 | 0.086 | 0.095 | 0.09 | 0.004 |
|  | mg kg^−1^ | PPS-2 | 0.063 | 0.075 | 0.067 | 0.006^***^ |
| Molybdenum | mg kg^−1^ | PPS-1 | BDL | BDL | BDL | --- |
|  | mg kg^−1^ | PPS-2 | BDL | BDL | BDL | -- |
| Titanium | mg kg^−1^ | PPS-1 | 99.785 | 99.812 | 99.792 | 0.016 |
|  | mg kg^−1^ | PPS-2 | 44.563 | 45.884 | 45.012 | 0.755^*^ |
| Boron | mg kg^−1^ | PPS-1 | 0.011 | 0.014 | 0.012 | 0.001 |
|  | mg kg^−1^ | PPS-2 | 0.005 | 0.006 | 0.005 | 0.000^ns^ |
| Selenium | mg kg^−1^ | PPS-1 | 0.004 | 0.006 | 0.005 | 0.001 |
|  | mg kg^−1^ | PPS-2 | BDL | BDL | BDL | -- |
| Vanadium | mg kg^−1^ | PPS-1 | BDL | BDL | BDL | -- |
|  | mg kg^−1^ | PPS-2 | BDL | BDL | BDL | -- |

BDL: Below detection limit; SD: Standard deviation; NS: Not specified; EC: Electrical conductivity; Asterisks indicate significant differences between the PPS-1 and PPS-2. Student’s t-test, two-tailed: *Highly significant at p < 0.001, **Significant at p < 0.01, ***Less significant at p < 0.05, nsNon-significant at p > 0.05.
