# Supplementary material for: Unraveling the shift in bacterial communities profile grown in sediments co-contaminated with chlorolignin waste of pulp-paper mill by metagenomics approach
Source: Front Microbiol. 2024 Mar 11;15:1350164. doi: 10.3389/fmicb.2024.1350164 (PMC10961449; doi:10.3389/fmicb.2024.1350164)
Supplement: Supplementary file 2 [file Table_2.docx]

**Table S2** Quantification of extracted metagenome recovered from sediment samples using NanoDrop

| **Sr. No.** | **Sample ID** | **Reading (ng/μl)** | **OD at A_260/280_** | **OD at A_260/230_** |
| --- | --- | --- | --- | --- |
| 1. | PPS-1 | 233.3 | 1.89 | 2.07 |
| 2. | PPS-2 | 261.8 | 1.88 | 2.10 |

OD: optical density
