# Supplementary material for: Unraveling the shift in bacterial communities profile grown in sediments co-contaminated with chlorolignin waste of pulp-paper mill by metagenomics approach
Source: Front Microbiol. 2024 Mar 11;15:1350164. doi: 10.3389/fmicb.2024.1350164 (PMC10961449; doi:10.3389/fmicb.2024.1350164)
Supplement: Supplementary file 3 [file Table_3.docx]

**Table** **S3** Bacterial diversity within the samples based on the operational taxonomic unit (OTUs) numbers recovered from the sediment samples. Shannon and Simpson indices are the estimator of alpha-diversity

| **Sample** | **Observed OTU** | **Shannon Index** | **Simpson Index** |
| --- | --- | --- | --- |
| PPS-1 | 1,249 | 7.99 | 0.987 |
| PPS-2 | 1,345 | 8.23 | 0.991 |

OTU: Operational taxonomic unit
