# Supplementary material for: Unraveling the shift in bacterial communities profile grown in sediments co-contaminated with chlorolignin waste of pulp-paper mill by metagenomics approach
Source: Front Microbiol. 2024 Mar 11;15:1350164. doi: 10.3389/fmicb.2024.1350164 (PMC10961449; doi:10.3389/fmicb.2024.1350164)
Supplement: Supplementary file 6 [file Table_6.docx]

**Table S6** Summary of the abundant bacterial community at different taxa identified in the samples

| **Taxa** | **PPS-1**  **(Relative abundance in %)** | **PPS-2**  **(Relative abundance in %)** |
| --- | --- | --- |
| Phylum | Proteobacteria (56.35) | Proteobacteria (56.35) |
| Class | Alphaproteobacteria (23.49) | Alphaproteobacteria (23.49) |
| Order | Rhodobacteriales (10.81) | Rhodobacteriales (10.81) |
| Family | Hydrogenophilaceae (8.00) | Rhodobacteraceae (8.00) |
| Genus | Thiobacillus (4.55) | Unclassified genus from Class ZB2 (4.55) |
| Species | Unclassified species (4.55) | Unclassified species from Class ZB2 (4.55) |
