# Supplementary material for: Unraveling the shift in bacterial communities profile grown in sediments co-contaminated with chlorolignin waste of pulp-paper mill by metagenomics approach
Source: Front Microbiol. 2024 Mar 11;15:1350164. doi: 10.3389/fmicb.2024.1350164 (PMC10961449; doi:10.3389/fmicb.2024.1350164)
Supplement: Supplementary file 7 [file Data_Sheet_1.docx]

Raw sequence data

FastQC, Trimmomatic v0.38

High quality read data

FLASH (v 1.2.11)

High quality read data

QIIME

GreenGenes database

(v 13_8)

OUT identification

RDP Classifier

QIIME

Taxonomic assignment of OTU

QIIME

Abundance estimation

QIIME

Comparative analysis

Rarefraction analysis

Diversity analysis

**Fig. S1** Flow chart for bioinformatic analysis of metagenomic DNA sequencing data recovered from sediment samples. OUT: Operational taxonomic unit; QIIME: Quantitative Insights Into Microbial Ecology; RDP: Ribosomal Database Project
