# Supplementary figures and images for: Unraveling the shift in bacterial communities profile grown in sediments co-contaminated with chlorolignin waste of pulp-paper mill by metagenomics approach
Source: Front Microbiol. 2024 Mar 11;15:1350164. doi: 10.3389/fmicb.2024.1350164 (PMC10961449; doi:10.3389/fmicb.2024.1350164)

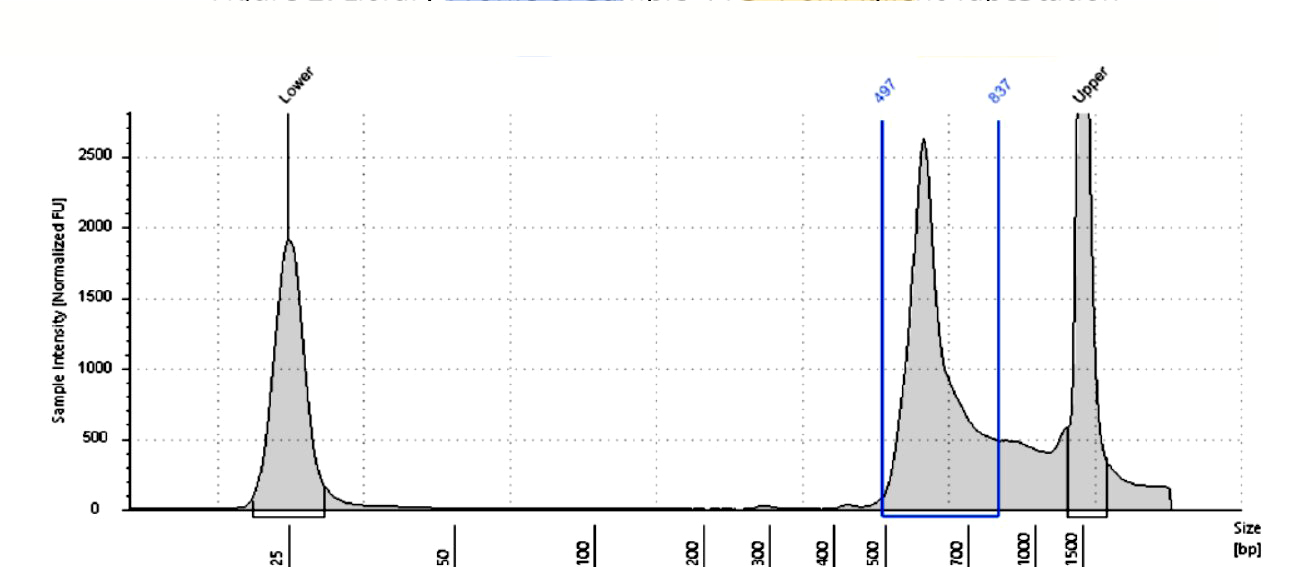

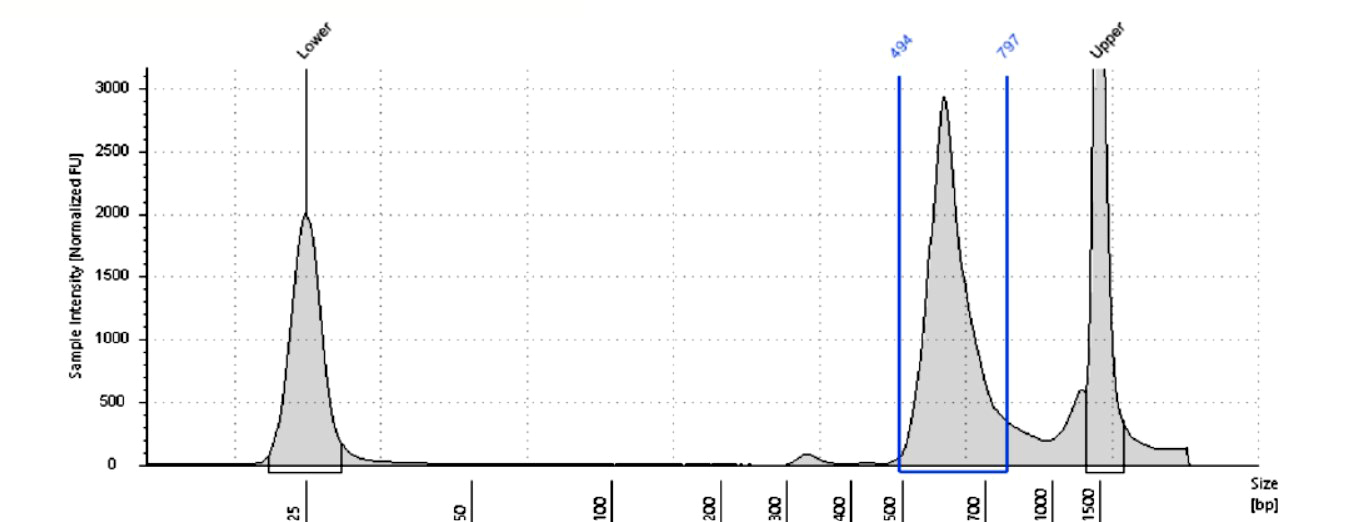


**(b)**

**(a)**

**Fig. S2** MiSeq library Profile of metagenome recovered from sediment samples. (a) PPS-1(b) PPS-2

Supplement: Supplementary file 8 [file Data_Sheet_2.docx]

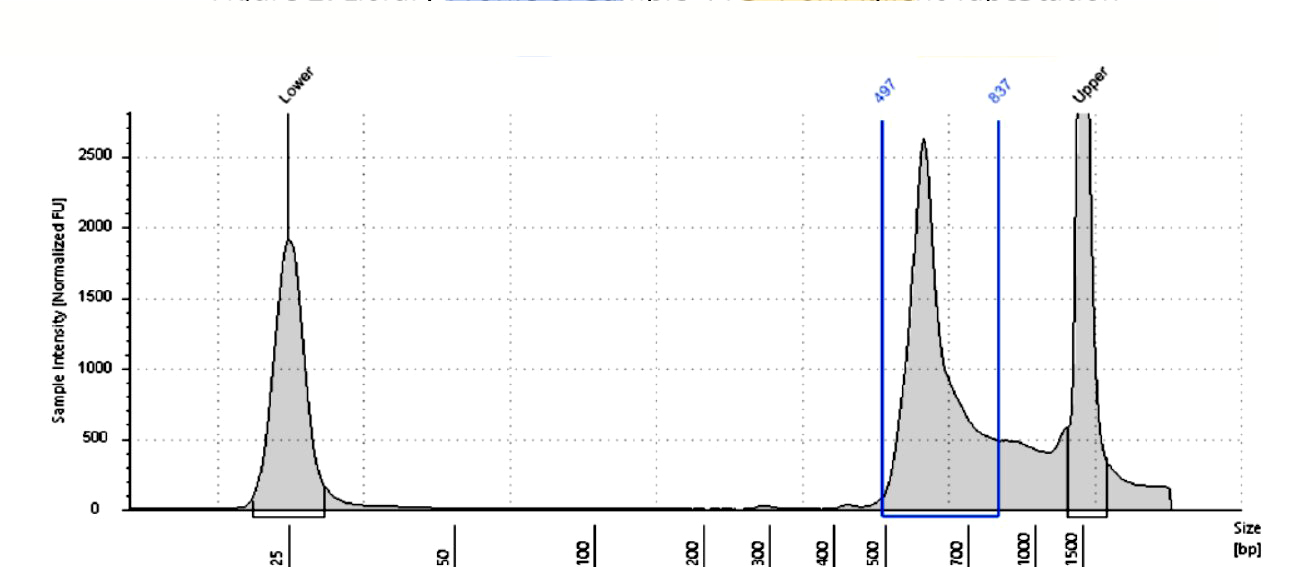

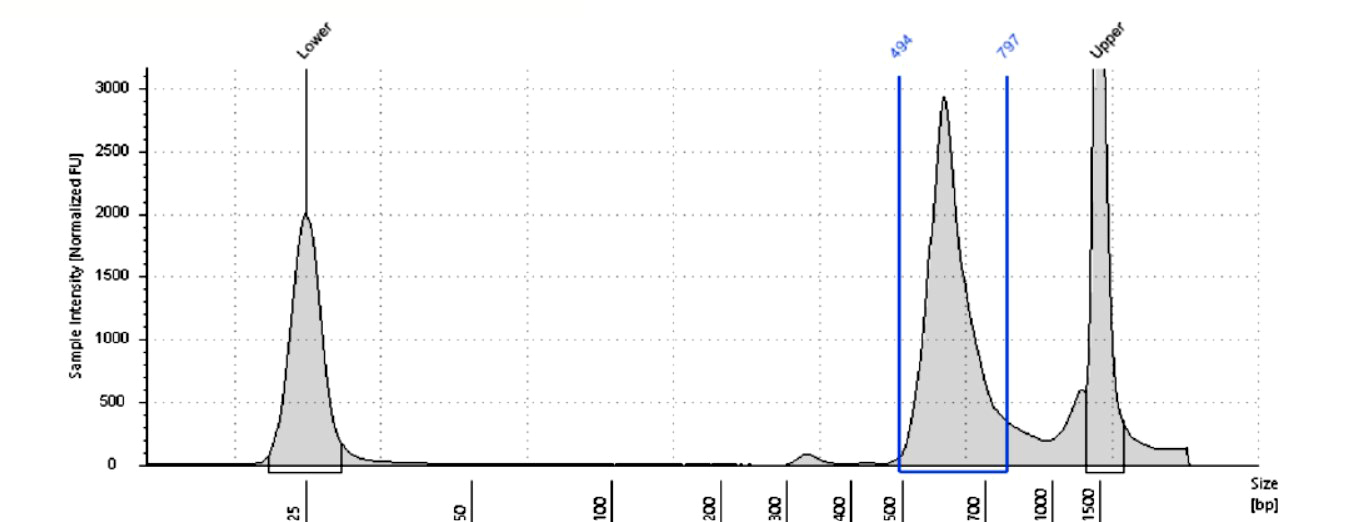


**(b)**

**(a)**

**Fig. S3** MiSeq library Profile of metagenome recovered from sediment samples. (a) PPS-1(b) PPS-2

Supplement: Supplementary file 9 [file Data_Sheet_3.docx]
